# Supplementary material for: Identification of sex determination genes and their evolution in Phlebotominae sand flies (Diptera, Nematocera)
Source: BMC Genomics. 2019 Jun 25;20:522. doi: 10.1186/s12864-019-5898-4 (PMC6593557; doi:10.1186/s12864-019-5898-4)
Supplement: Supplementary file 3 — Figure S10. Manually-curated P. perniciosus transformer gene model. Figure S11. Manually-curated P. papatasi transformer gene model. Figure S12. Manually-curated P. bergeroti transformer gene model. Figure S13. Manually-curated P. duboscqi transformer gene model. (PDF 349 kb) [file 12864_2019_5898_MOESM3_ESM.pdf]

P. perniciosus tra gene model

Clone_G1_Length=1725

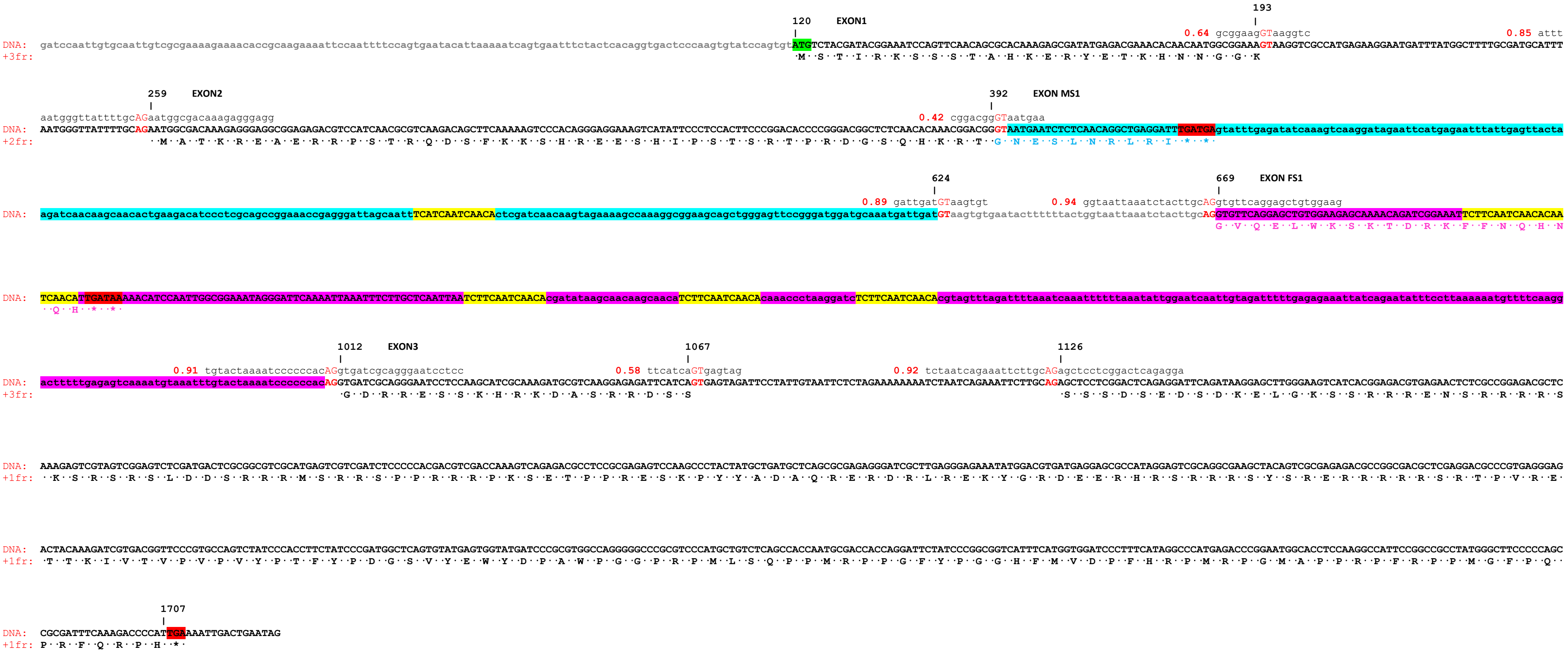

Figure S10. Manually-curated *P. perniciosus transformer* gene model. Exonic sequences are indicated by black cases; black upper cases indicate coding sequences. Intronic sequences are indicated by gray lower cases. Start and stop codons are highlighted in green and red, respectively. In yellow boxes, putative TRA/TRA-2 binding sites. Exon start/end positions in the scaffold/s are indicated. Intron boundaries were predicted using transcripts vs genome alignments and confirmed by *de novo* prediction with Berkeley BDGP Splice Site Prediction Tool with default parameters (http://www.fruitfly.org/seq_tools/splice.html); prediction scores are indicated in red. Azure box indicates the male-specific exon 1 sequence; Purple box indicates the famel-specific exon 1 sequence.

P. papatasi tra gene model

```
Scaffold2305_Length=23430

                                                                                                    EXON1                                      5365
                                                                                               5295                                             |
                                                                                                |                                     0.49 gcggaagGTtagagc
DNA:  agaggagtagccgcaaaatccgcaaaacagtgaaaaaattgtcaatataccagtgaaattggttaatatatttttatatctattccaagttgttggtaaaagtgtccagtgtgtatctATGATTAAAAAACCTAGCTCCAAGACGCACAAGGAAAAATATGAGGGAAAGTACAAAACAAATGGCGGAAGgttagagccttgtctgactaacaatggcttccggaaaattcccagaaatgc
+3fr:                                                                                                                             ·M··I··K··K··P··S··S··K··T··H··K··E··K··Y··E··G··K··Y··K··T··N··G··G··R

                       5444      EXON2                                                                                                                                             5634
                        |                                                                                                                  ------------------>                       |
 0.97 ttcaatgattttgctttgcAGatcggcatcgaagagggaag                                                                                                                       0.30 aggacggGTaatggc
DNA:  tgcaaattcaatgattttgctttgcagATCGGCATCGAAGAGGGAAGCAGAGAGACATCCATCAACACATGAAGACACCATCAAAAAATCCCATCATCATCGTGATGAATTAGGAAAGAAAGATGAAAGAAAGACTTATGAGGACGAATTTCCCAAACTTGGGCATGTTCCATCTACATCACGGGATTCCCGGGACGGCTTTCAACACAAAAGGACGGgtaatggcatgaagaaaccttct
+3fr:                            ··S··A··S··K··R··E··A··E··R··H··P··S··T··H··E··D··T··I··K··K··S··H··H··H··R··D··E··L··G··K··K··D··E··R··K··T··Y··E··D··E··F··P··K··L··G··H··V··P··S··T··S··R··D··S··R··D··G··F··Q··H··K··R··T··

DNA:  atcaatgtcaacactagaagaacatgtcacaagtcagagaatttgatgagtatttttttgaaaataagagattagaatattttgtgaataaattttcatgagattcttgagactttaaagtaaaagagagatcatcaagatgtccaaataatctagagagcagaTCATCAATCAACAcagacgtttctgagagctgaacaacgaggtgtaggagctgaggagacgaatcgtttctaggatag

DNA:  gaaaaagtaagtttgaaacattttccgtaactttatctactttcagaataatccgggagtttagcagaacaacgccaagggatatcttcctctcaagattCTTTCAATCAACAttcagcaaataccaattggcgggagtgtgggtagccaaaaattaattcaatacgaaTCTTCAATCAACAagttGCCCCAATCAACAcccttaaattTCTTCAATCAACAccatttcctggaggatatt

                                                                                                                       6275      EXON3                                 6330
                                                                                                                        |                                                |
                                                                                              0.77 ttttgcaataaactcccacAGgtgatggcaaggaatcctct                  0.77 ttcttcaGTaagtaa                     0.55 gattgagag
DNA:  TCTTCAATCAACAcgtagattttgctcaaataacttaatttttttgtaaaattgttattaaagaaaagaattaactttgttttgtggagttatttaaagaaattgtcatgtaaattttgcaataaactcccacagGTGATGGCAAGGAATCCTCTAAGCATCGAAGAGGTGCTGCCAAGAGAGATTCTTCAgtaagtaaaaaagtacctaaaaccgagagtaagagagatttgattgagag
+1fr:                                                                                                                                   G··D··G··K··E··S··S··K··H··R··R··G··A··A··K··R··D··S··S·

            6393      EXON4
             |
      agttttttgtAGaattcttcagagactgacga
DNA:  agttttttgtagAATTCTTCAGAGACTGACGATGAATCCCTGAAATCTTCGAGGAAAAGGCCAGAATCTCGCCGGAGACGATCAAAAAGTCGTAGCCGAAGTCTCGATGACTCGTACAGGAGACGTCAAGCTTCCACGAGTCGTCGATCTTCGCCACGGCGTCGGCCAAAGTCCGTTTCTCCTCCGCGTGAGAAAGTTCCCTACTATGCAGATGCCCAGCGTGAGAGGGATCGCCTGAGGG
+3fr:              ·N··S··S··E··T··D··D··E··S··L··K··S··S··R··K··R··P··E··S··R··R··R··R··S··K··S··R··S··R··S··L··D··D··S··Y··R··R··R··Q··A··S··T··S··R··R··S··S··P··R··R··R··P··K··S··V··S··P··P··R··E··K··V··P··Y··Y··A··D··A··Q··R··E··R··D··R··L··R··

                                                                                                                                                   <------------------
DNA:  AGAAGTATGGAAGTGACGGGAGGACTAGGAGGAGGAGCAGCAGCCGCGAGAGACGTAGAAGACGCTCTAGAGATAGGAGATCCAAGACTCCAGTCCGAGAAACCACAAAAATTGTCACAGTTCCTGTTCCTGTTCCCGTCTACCCATCAATATATCCGGATGGTTCCATGTATGACTGGTACGATCCTTCTTGGCAAACAGGACTCTTTTCTCAGGCTGTCATGCCTCCAAGACACATTCC
+3fr: E··K··Y··G··S··D··G··R··T··R··R··R··S··S··S··R··E··R··R··R··R··R··S··R··D··R··R··S··K··T··P··V··R··E··T··T··K··I··V··T··V··P··V··P··V··P··V··Y··P··S··I··Y··P··D··G··S··M··Y··D··W··Y··D··P··S··W··Q··T··G··L··F··S··Q··A··V··M··P··P··R··H··I··P

                                                                                                                                 7007
                                                                                                                                  |
DNA:  ACGCCCAATGCGACCTCATGCCCCATTCCCTACTTCACCCTTCATGGTGGATCCTTTTCATCGGCCAATGAGGCCAGGAATGCCACCTCCAAGACCCTTTCGGCCTCCCCAAGGATTCCCTCCTCCCCGATTCCACAGACCCCATTGAaatgaaagtctgaaaatactaattattgtgagcaaaaagccataagtttattaagaataaaatgtattgaatttttt
+3fr:  ··R··P··M··R··P··H··A··P··F··P··T··S··P··F··M··V··D··P··F··H··R··P··M··R··P··G··M··P··P··P··R··P··F··R··P··P··Q··G··F··P··P··P··R··F··H··R··P··H··*·
```

Figure S11. Manually-curated *P. papatasi transformer* gene model. Exonic sequences are indicated by black cases; black upper cases indicate coding sequences. Intronic sequences are indicated by gray lower cases. Start and stop codons are highlighted in green and red, respectively. In yellow boxes, putative TRA/TRA-2 binding sites. Exon start/end positions in the scaffold/s are indicated. Intron boundaries were predicted using transcripts vs genome alignments and confirmed by *de novo* prediction with Berkeley BDGP Splice Site Prediction Tool with default parameters (http://www.fruitfly.org/seq_tools/splice.html); prediction scores are indicated in red. In purple boxes are indicated the positions of the primers utilized for *PpatraF* amplification.

P. bergeroti tra gene model

```
Contig_32222_414377_7522_Length=2705

DNA:  caaatatctggcggctttcccgaaatgaaacgtgggaggtttttttttcactttaatatcacgagactctttgtaacacacctcaataaacacctgaaacagacacaagatgtgacttcagacactgggtcaaggtttaacgaatatttaaaggcaatttttattgaattcagtgtgccagatgactttccagataagaaaatttagagatttctcaaatgaattcccttccggaaaccc

DNA:  atccgccattgctttcttcccgtcgttgataaaccacgccacaatgtccactcaccgacttgcagcaaaatgtcccccgacgattatttatcacactcacaattataaatagagtgaaaggaggaaaaaggtcagagatttgagggaaaatataaatgcaaatgtagcgagaaatgtgacaagtctgcggccatgaagagactccactttttccacctcaaatcacactctgaataccac

DNA:  aaatttttcacttatttcaaatgcagaagatacttggacactttaatttgaggttcattgtatttttttccactaatttttagtatttttaggcgaaaaattgcatttattccgcagttgcatattccgatttctgcacctttttacttgtcaaaccagctgtttttttagccaccaggcggcgctaattgccatattgtgaaataaaaaaaaaggcctattagcaagaggagtagccgcaaaa

      EXON1  827  897
      0.82 gcggaagGTtagtgt      0.97 tcaatgatt
DNA:  tccgcaaaactgtgaaaaaattgacaaattaccagtgaaagttacttaaatattatttttatatctattccaagttgttggtaaaagtatccagtgtgtatctATGATTAAAAAACCTAGCTCCAGGACGCACAAGGAAAAATATGAGGGAAAATACAAAACAAATGGCGGAAGgttagtgtatagacagcttaacaatggcttccggaaaattcccagaaatgctgcaaattcaatgatt
+3fr: ·M··I··K··K··P··S··S··R··T··H··K··E··K··Y··E··G··K··Y··K··T··N··G··G··R

      977  EXON2  1167
      tttgcagatcAGcatcgaagagggagg      0.41 agaacggGTaatggc
DNA:  tttgctttgcagATCAGCATCGAAGAGGGAGGCAGAGAGACATCCATCAACACATGAAGACACCATCAAAAAATCCCATCATCATCGTGATGAATTAGGAAAGAAAGATGAAAGGAAGTCTTATGAGGATGATTTTCCCAAACTTGGGCATGTTCCATCTACATCACGGGATACCCGGGACGGCTTTCAACACAAAAGAACGGgtaatggcatgaagaaatcttctatcaatgtcaacact
+1fr: ··S··A··S··K··R··E··A··E··R··H··P··S··T··H··E··D··T··I··K··K··S··H··H··H··R··D··E··L··G··K··K··D··E··R··K··S··Y··E··D··D··F··P··K··L··G··H··V··P··S··T··S··R··D··T··R··D··G··F··Q··H··K··R··T··

DNA:  agaagaacatgtcacaagtcagagaattttgatgagtattttattgaaataaagagattagaatattttgtgaaataattttcatgagattcttgagattttaaagtaaaagagagatcatcaagatgcccaaataatctagagagcagaTCATCAATCAACAcagacgtttctgagagctgaacaacgatgtgtaggagctgaggagaagaatcgtttctaggattgaaaaaagtaagtt

DNA:  tgaaatattttccgtaactttatctactttcagaataatccaggagcttagcagaacgccaagggatatttttctctcaagattCTTTCAATCAACAttcagcaaattccaattggcgggagagtgggtagccaaaaagtaattcaatacgaaTCTTCAATCAACAagttGCCCCAATCAACAcacttaaattTCTTCAATCAACAcaatttcctgaaggatattTCTTCAATCAACAcgt

      1807  EXON3  1862  1926
      0.85 ttgcaataaactcccccacAGgtgatggcagggaatcctct      0.77 ttcttcaGTaagtaa      0.69 gatttagggagtttttttgtAGaat
DNA:  agattttgctcaattaacttttttttgaaaaattttattaaagaaaagaattaactttgttttctggagttatttaaagaaattgtcatgtaaattttgcaataaactcccccacagGTGATGGCAGGGAATCCTCTAAGCATCGAAGAGGTGCTGCCAAGAGAGATTCTTCAgtaagtaaaaagaaaagtacttaaaatcgggtgtagagatttgatttagggagtttttttgtagAAT
+2fr: G··D··G··R··E··S··S··K··H··R··R··G··A··A··K··R··D··S··S·      ·N·

      tcttcagagactgacag
DNA:  TCTTCAGAGACTGACAGTGAATCCCTCAAATCTTCGAGGAAAAGGCCAGAATCTCGCCGGAGACGATCAAAAAGTCGTAGCAGAAGTCTCGATGACTCGTACAGGAGACGTCAGACCTCCACGAGTCGTCGATCTTCACCACGACGCCGGCCAAAGTCTGTTTCTCCACCGCGGGAGAAAGTGCCCTACTATGCAGATGCCCAGCGTGAGAGGGATCGCCTGAGAGAGAAGTATGGAAGTG
+3fr: ·S··S··E··T··D··S··E··S··L··K··S··S··R··K··R··P··E··S··R··R··R··R··S··K··S··R··S··R··S··L··D··D··S··Y··R··R··R··Q··T··S··T··S··R··R··S··S··P··R··R··R··P··K··S··V··S··P··P··R··E··K··V··P··Y··Y··A··D··A··Q··R··E··R··D··R··L··R··E··K··Y··G··S··

DNA:  ATGGGAGGATGAGGAGGAGGAGCAGCAGCCGCGAGAGACGCAGAAGACGCTCTAGAGATAGAAGATCCAAGACTCCAGTCCGAGAAACCACAAAAATTGTCACAGTTCCTGTTCCTGTTCCCGTCTACCCATCAATTTATCCAGATGGTTCCATGTATGACTGGTACGATCCCTCTTGGCAAACAGGGCTCTTTCCACAGGGTGTAATGCCTCCAAGACACATTCCACGCCCAATGCGACC
+3fr: D··G··R··M··R··R··R··S··S··S··R··E··R··R··R··R··R··S··R··D··R··R··S··K··T··P··V··R··E··T··T··K··I··V··T··V··P··V··P··V··P··V··Y··P··S··I··Y··P··D··G··S··M··Y··D··W··Y··D··P··S··W··Q··T··G··L··F··P··Q··G··V··M··P··P··R··H··I··P··R··P··M··R··P

      2540
DNA:  TCATGCCCCATTCCCTACTTCACCCTTCATGGTGGATCCTTTTCATCGGCCAATGAGGCCAGGAATGCCACCTCCAAGACCCTTTCGACCTCCCCAAGGATTCCCTCCTCCCCGATTCCACAGACCCCATTGAaatacaaagtctgaaaatactattgtgagcaaaagccattttaagaataaaatgtattgaatttttattgtttttttttttagagaggaattctcgttatagagaaat
+3fr: ··H··A··P··F··P··T··S··P··F··M··V··D··P··F··H··R··P··M··R··P··G··M··P··P··P··R··P··F··R··P··P··Q··G··F··P··P··P··R··F··H··R··P··H··*·

DNA:  tattaataggggaaagtggggcatatttgaaagtcgagcacctttgaaattggg
```

Figure S12. Manually-curated *P. bergeroti transformer* gene model. Exonic sequences are indicated by black cases; black upper cases indicate coding sequences. Intronic sequences are indicated by gray lower cases. Start and stop codons are highlighted in green and red, respectively. In yellow boxes, putative TRA/TRA-2 binding sites. Exon start/end positions in the scaffolds are indicated. Intron boundaries were predicted using transcripts vs genome alignments and confirmed by *de novo* prediction with Berkeley BDGP Splice Site Prediction Tool with default parameters (http://www.fruitfly.org/seq_tools/splice.html); prediction scores are indicated in red.

P. duboscqi tra gene model

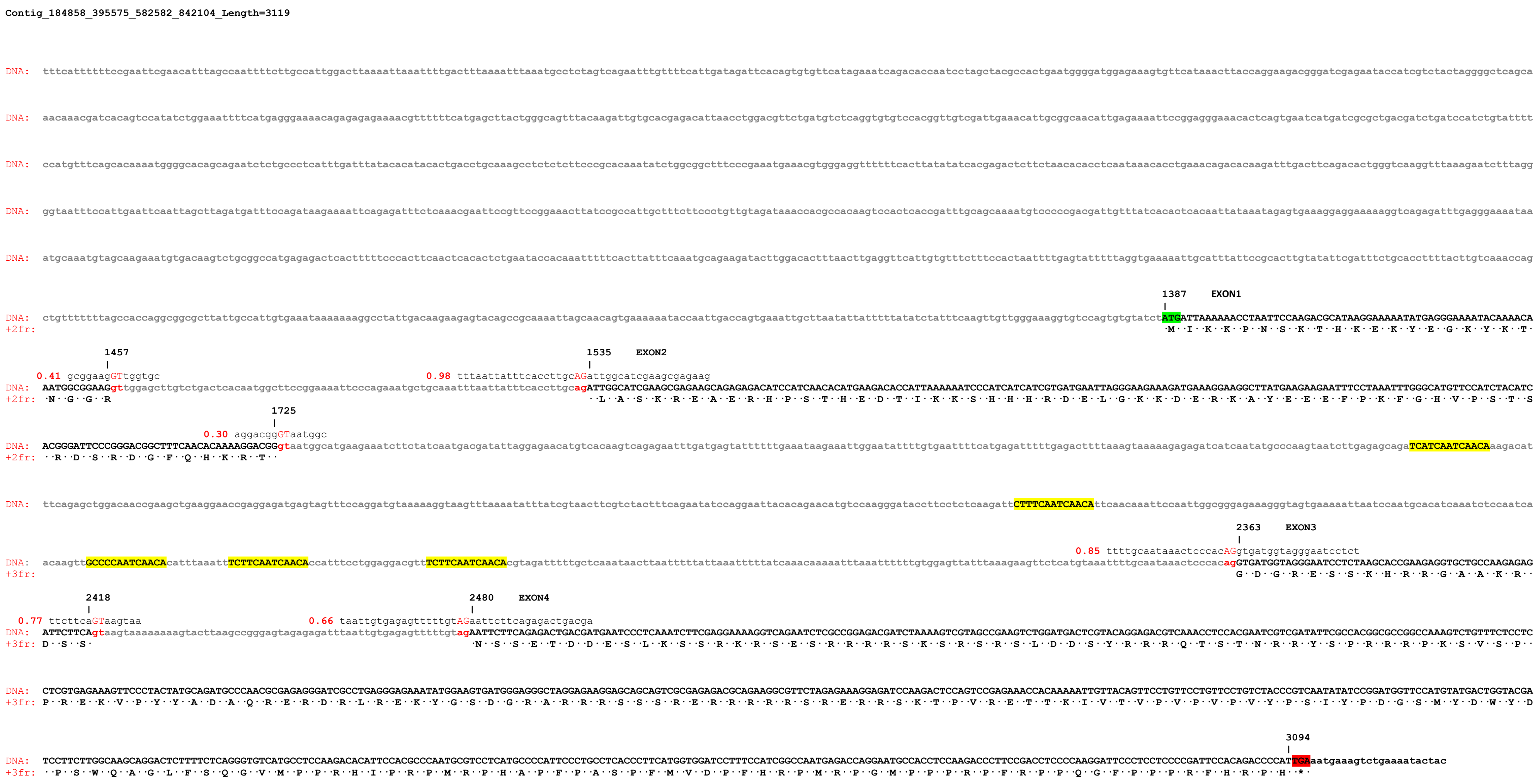

Figure S13. Manually-curated *P. duboscqi transformer* gene model. Exonic sequences are indicated by black cases; black upper cases indicate coding sequences. Intronic sequences are indicated by gray lower cases. Start and stop codons are highlighted in green and red, respectively. In yellow boxes, putative TRA/TRA-2 binding sites. Exon start/end positions in the scaffolds are indicated. Intron boundaries were predicted using transcripts vs genome alignments and confirmed by *de novo* prediction with Berkeley BDGP Splice Site Prediction Tool with default parameters (http://www.fruitfly.org/seq_tools/splice.html); prediction scores are indicated in red.
