# Supplementary material for: Identification of sex determination genes and their evolution in Phlebotominae sand flies (Diptera, Nematocera)
Source: BMC Genomics. 2019 Jun 25;20:522. doi: 10.1186/s12864-019-5898-4 (PMC6593557; doi:10.1186/s12864-019-5898-4)
Supplement: Supplementary file 4 — Figure S14. Multiple sequence alignment of TRA proteins in Phlebotomus spp. (PDF 251 kb) [file 12864_2019_5898_MOESM4_ESM.pdf]

```
PpeTRA      MSTIRKSSSTAHKERYETK-HNNGGK|MATKREAERRPSTRQDSFKKSHRE----------     49
PduTRA      --MIKKPNSKTHKEKYEGKYKTNGGR|LASKREAERHPSTHEDTIKKSHHHRDELGKKDER     58
PpaTRA      --MIKKPSSKTHKEKYEGKYKTNGGR|SASKREAERHPSTHEDTIKKSHHHRDELGKKDER     58
PbeTRA      --MIKKPSSRTHKEKYEGKYKTNGGR|SASKREAERHPSTHEDTIKKSHHHRDELGKKDER     58
               *:* .* :***:** * :.***: *:******:***::*::****:.

PpeTRA      ---------ESHIPSTSRTPRDGSQHKRTGDRRESSKHRKDASRRDSSSSSDSEDSDKEL     100
PduTRA      KAYEEEFPKFGHVPSTSRDSRDGFQHKRTGDGRESSKHRRGAAKRDSSNSSETD---DES     115
PpaTRA      KTYEDEFPKLGHVPSTSRDSRDGFQHKRTGDGKESSKHRRGAAKRDSSNSSETD---DES     115
PbeTRA      KSYEDDFPKLGHVPSTSRDTRDGFQHKRTGDGRESSKHRRGAAKRDSSNSSETD---SES     115
                      .*:*****  *** ******* :******:.*::****.**:::   .*

                                                            DIPTERA domain
PpeTRA      GKSSRRRENSRRRRSKSRSRSLDDSRRRM----SRRSPPRRRPKSETPPRESKPYYADAQ     156
PduTRA      LKSSRKRSESRRRRSKSRSRSLDDSYRRRQTSTNRRYSPRRRPKSVSPPREKVPYYADAQ     175
PpaTRA      LKSSRKRPESRRRRSKSRSRSLDDSYRRRQASTSRRSSPRRRPKSVSPPREKVPYYADAQ     175
PbeTRA      LKSSRKRPESRRRRSKSRSRSLDDSYRRRQTSTSRRSSPRRRPKSVSPPREKVPYYADAQ     175
             ****:* :*************** **     .**  ******* :****. *******

PpeTRA      RERDRLREKYGRDEERHRSR--RRSYSRERRRRRSRTPVRETTKIVTV--PVPVYPTFYP     212
PduTRA      RERDRLREKYGSDGRARRRSSSRERRRRRSRERRSKTPVRETTKIVTVPVPVPVYPSIYP     235
PpaTRA      RERDRLREKYGSDGRTRRRSSSRERRRRRSRDRRSKTPVRETTKIVTVPVPVPVYPSIYP     235
PbeTRA      RERDRLREKYGSDGRMRRRSSSRERRRRRSRDRRSKTPVRETTKIVTVPVPVPVYPSIYP     235
            *********** *  . :*    *.   *. * ***:************  ******::**

PpeTRA      DGSVYEWYDPAWPGGPRPM------LSQPPMRPPGFYPGGHFMVDPFHRPMRPGMAPPRP     266
PduTRA      DGSMYDWYDPSWQAGLFSQGVMPPRHIPRPMRPHAPFPASPFMVDPFHRPMRPGMPPPRP     295
PpaTRA      DGSMYDWYDPSWQTGLFSQAVMPPRHIPRPMRPHAPFPTSPFMVDPFHRPMRPGMPPPRP     295
PbeTRA      DGSMYDWYDPSWQTGLFPQGVMPPRHIPRPMRPHAPFPTSPFMVDPFHRPMRPGMPPPRP     295
            ***:*:****:*  *             ****  . :*  . ************* ****

PpeTRA      FRPPMGFPQPRFQRPH  282
PduTRA      FRPPQGFPPPPRFHRPH  311
PpaTRA      FRPPQGFPPPPRFHRPH  311
PbeTRA      FRPPQGFPPPPRFHRPH  311
            **** *** ***:***
```

Figure S14. Multiple sequence alignment of TRA proteins in *Phlebotomus* spp. Sequence alignment of TRA proteins of *P. perniciosus* (PpeTRA), *P. duboscqi* (PduTRA), *P. papatasi* (PpaTRA) and *P. bergeroti* (PbeTRA). The DIPTERA domain is highlighted in grey. The position of the sex-specifically regulated splicing site between exon2 and exon3 is indicated by the pipe symbol (|). Gaps were introduced in the alignment to maximize similarity. The protein sequences alignment was performed using the Clustal-Omega software (1.2.4).
