# Supplementary material for: Identification of sex determination genes and their evolution in Phlebotominae sand flies (Diptera, Nematocera)
Source: BMC Genomics. 2019 Jun 25;20:522. doi: 10.1186/s12864-019-5898-4 (PMC6593557; doi:10.1186/s12864-019-5898-4)
Supplement: Supplementary file 5 — Figure S15. Multiple alignment of tra introns in Phlebotomus spp. (PDF 572 kb) [file 12864_2019_5898_MOESM5_ESM.pdf]

A)

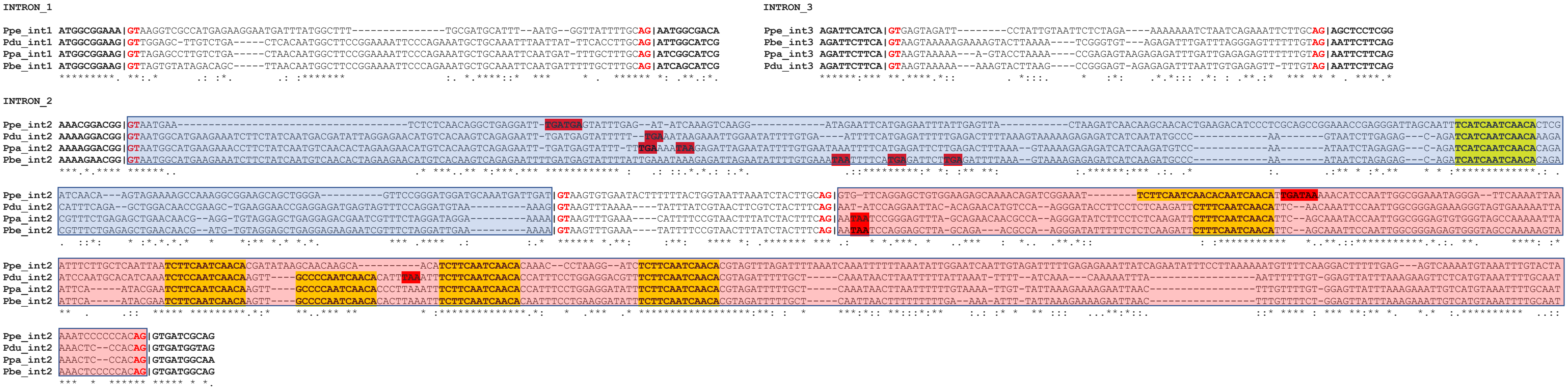

B)

Intron 1							
Species	5' Donor SS	Identity	BDGP SS Pred. Score	3' Acceptor SS	Identity	Cons. n° of py = 8.01+/-1.98	BDGP SS Pred. Score
	GTRAGY			YYYYYYYYYYYYNYAG		n° o py	
Ppe	GTAAGG	5/6	0.64	ATGGGTTATTTTGCAG	10/16	7	0.85
Ppa	GTTAGA	4/6	0.49	TGATTTTGCTTTGCAG	13/16	9	0.97
Pbe	GTTAGT	5/6	0.82	GATTTTTGCTTTGCAG	13/16	9	0.98
Pdu	GTTGGA	3/6	0.41	TTATTTCACCTTGCAG	14/16	10	0.97

Intron 2							
Species	5' Donor SS	Identity	BDGP SS Pred. Score	3' Acceptor SS	Identity	Cons. n° of py = 8.01+/-1.98	BDGP SS Pred. Score
	GTRAGY			YYYYYYYYYYYYNYAG		n° o py	
Ppe	GTAATG	5/6	0.42	TAAAATCCCCCCACAG	12/16	8	0.91
Ppa	GTAATG	5/6	0.30	CAATAAACTCCCACAG	11/16	7	0.77
Pbe	GTAATG	5/6	0.41	ATAAACTCCCCCACAG	12/16	8	0.85
Pdu	GTAATG	5/6	0.30	CAATAAACTCCCACAG	11/16	7	0.85

Intron 3							
Species	5' Donor SS	Identity	BDGP SS Pred. Score	3' Acceptor SS	Identity	Cons. n° of py = 8.01+/-1.98	BDGP SS Pred. Score
	GTRAGY			YYYYYYYYYYYYNYAG		n° o py	
Ppe	GTGAGT	6/6	0.58	TCAGAAATTCTTGCAG	11/16	7	0.92
Ppa	GTAAGT	6/6	0.77	AGAGAGTTTTTTGTAG	10/16	6	0.55
Pbe	GTAAGT	6/6	0.77	AGGGAGTTTTTTGTAG	10/16	6	0.69
Pdu	GTAAGT	6/6	0.77	GTGAGAGTTTTTGTAG	10/16	6	0.66

ms1_male-specific			
Species	5' Donor SS	Identity	BDGP SS Pred. Score
	GTRAGY		
Ppe	GTAAGT	6/6	0.89
Ppa	GTAAGT	6/6	0.96
Pbe	GTAAGT	6/6	0.96
Pdu	GTAAGT	6/6	0.99

fs1_female-specific				
Species	3' Acceptor SS	Identity	Cons. n° of py = 8.01+/-1.98	BDGP SS Pred. Score
	YYYYYYYYYYYYNYAG		n° o py	
Ppe	TTAAATCTACTTGCAG	12/16	8	0.94
Ppa	CTTCGTCTACTTTCAG	14/16	10	0.91
Pbe	CTTTATCTACTTTCAG	14/16	10	0.91
Pdu	CTTTATCTACTTTCAG	14/16	10	0.84

Figure S15. Multiple alignment of *tra* introns in *Phlebotomus* spp. A) All introns exhibit GT/AG consensus terminal dinucleotides (in red). Exonic flanking sequences are indicated by bold cases; exon/intron boundaries are indicated by the pipe | symbol. Azure box represents the male-specific ms1 exon. Pink box represents the female-specific fs1 exon. In frame stop codons are highlighted in red. Putative TRA/TRA-2 binding sites are highlighted in yellow. Intron boundaries were predicted using transcripts vs genome alignments for *P. perniciosus* and *P. papatasi* and *de novo* predicted by Berkeley BDGP Splice Site Prediction Tool with default parameters (http://www.fruitfly.org/seq_tools/splice.html) for *P. bergeroti* and *P. duboscqi*. **B)** Splicing site (SS) score values and consensus match for all the splicing sites are reported. Bold letters indicate match with the consensus sequences. Strong SS were highlighted in green and weak SS in red. Consensus number of pyrimidine of 3' Acceptor SS in sand flies has been calculated by the tabulation of 25.000 random acceptor SS extracted from the *P. papatasi* PpapI1.4 gene set using the Biomart tool of VectorBase. (M=A or C; W=A or T; R=A or G; Y=C or T; N=any nucleotide).
