# Supplementary material for: Identification of sex determination genes and their evolution in Phlebotominae sand flies (Diptera, Nematocera)
Source: BMC Genomics. 2019 Jun 25;20:522. doi: 10.1186/s12864-019-5898-4 (PMC6593557; doi:10.1186/s12864-019-5898-4)
Supplement: Supplementary file 6 — : Figure S16. tra gene expression at adult stage in P. papatasi. (PDF 137 kb) [file 12864_2019_5898_MOESM6_ESM.pdf]

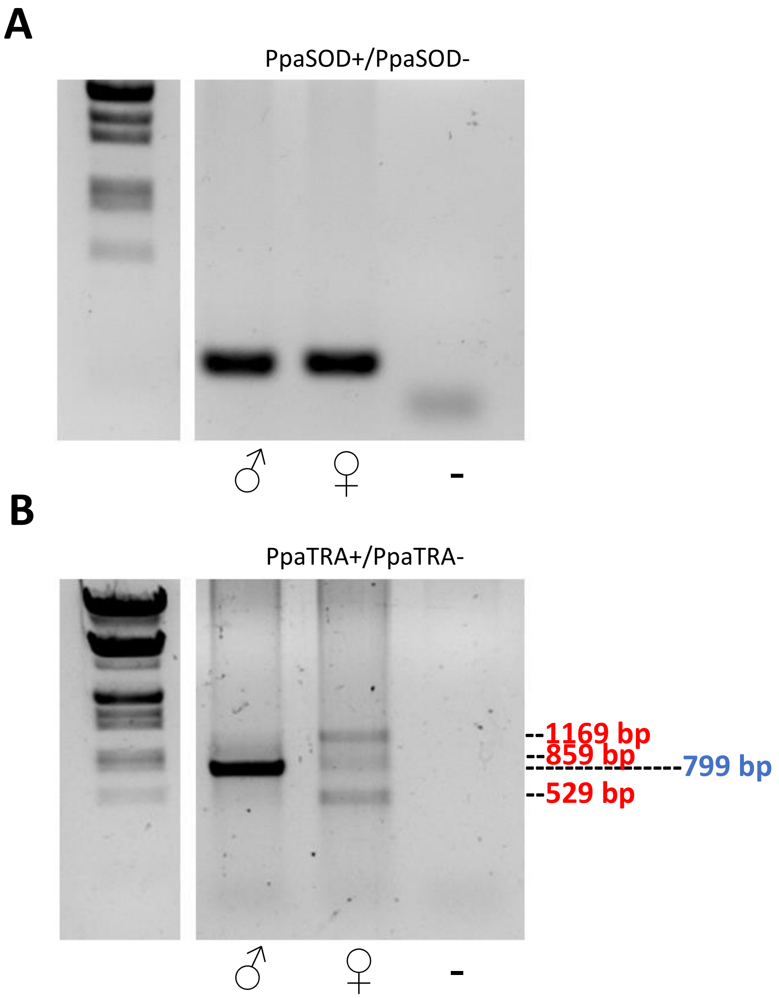

Figure S16. *tra* gene expression at adult stage in *P. papatasi*. RT-PCR analysis on total RNA extracted from adult males and females of *P. papatasi*. (A) Control RT-PCR with primer pair PpaSOD+/PpaSOD-. (B) RT-PCR with primer pair PpaTRA+/PpaTRA-. λDNA digested with EcoRI and HindIII endonucleases (Marker III – Sigma Aldrich) was utilized as molecular weight marker.
