# Supplementary material for: Identification of sex determination genes and their evolution in Phlebotominae sand flies (Diptera, Nematocera)
Source: BMC Genomics. 2019 Jun 25;20:522. doi: 10.1186/s12864-019-5898-4 (PMC6593557; doi:10.1186/s12864-019-5898-4)
Supplement: Supplementary file 9 — Figure S21. Manually-curated P. papatasi doublesex gene model. Figure S22. Manually-curated L. longipalpis doublesex gene model. Figure S23. Manually-curated P. papatasi fruitless gene model. Figure S24. Manually-curated L. longipalpis fruitless partial gene model. Figure S25. Comparison of genomic structures of dipteran dsx genes. Figure S26. Comparison of genomic structures of dipteran fru genes. (PDF 283 kb) [file 12864_2019_5898_MOESM9_ESM.pdf]

P. papatasi dsx gene model

Scaffold7_Length=1359615

994630 EXON1

DNA: ctaaattctctgtatatctagactaagtgtcagtcttcggttaaaagttcaattaagtgggacgctgttgATGGTGTCGGAGCACAATACGTGGAGCGACGTGATGTCCAATTCGGACCTCACAGACTCGAAAAACGAGTTGTGCGGAGGAGCCTCGAGCTCCAGCGGCAACTCATTGAGCCCGAGGACGCCACCAAATTGCGCCCGCTGCCGCAATCACGGCCTCAAGATAGCCCTTAA
+2fr: ·M··V··S··E··H··N··T··W··S··D··V··M··S··N··S··D··L··T··D··S··K··N··E··L··C··G··G··A··S··S··S··S··G··N··S··L··S··P··R··T··P··P··N··C··A··R··C··R··N··H··G··L··K··I··A··L··K

DNA: GGGTCACAAACGCTACTGCAAGTACCGTTACTGCAACTGCGAAAAGTGTCGTCTAACGGCCGAACGTCAGCGGGTGATGGCCTTGCAGACGGCCCTAAGACGTGCTCAGGCTCAGGACGAGGCACGTCAGATCTCCGCGGATGAGGTGCCACCGACGCCACCGCCAGCCCCGTCCATTTGCACCCTTGCCACTCCTAAGAGTCAGTGCGATAGCGAACCACCGGCACCCATTACGGTGCC
+2fr: ··G··H··K··R··Y··C··K··Y··R··Y··C··N··C··E··K··C··R··L··T··A··E··R··Q··R··V··M··A··L··Q··T··A··L··R··R··A··Q··A··Q··D··E··A··R··Q··I··S··A··D··E··V··P··P··T··P··P··P··A··P··S··I··C··T··L··A··T··P··K··S··Q··C··D··S··E··P··P··A··P··I··T··V··P

995176

0.91 actgcaaGTaagtac

DNA: CACACCGGCACGTTCCCTAGAGGGTTCCTGCCACTCATCATCGGCCACACCCTGTTCTGCTTCCGGAGAACCCATCTCCGTGCCATCCAGCCGCAAATTGGCCCCAGTCAATCCAACCATCAGAAATCCAACTGCAAgtaagtaccattcaaattatccttactacgtatatggtaccctctacaacagctaccctacttatggccacatacaccaaaaaaaaaatataagcttaatcct
+2fr: ··T··P··A··R··S··L··E··G··S··C··H··S··S··S··A··T··P··C··S··A··S··G··E··P··I··S··V··P··S··S··R··K··L··A··P··V··N··P··T··I··R··N··P··T··A··

----////----

1114154 EXON2 1114294

0.90 ttcctcttgtcacgctttcAGgtcaaagctccgaagtcttc 0.90 gaagaagGTgagatg

DNA: cttgattttaatgttcaactcttcctcttgtcacgctttcagGTCAAAGCTCCGAAGTCTTCTTTGACAATTGCCAGAAATTGCTGGACAAATTCAATTATCCTTGGGAACTGATGCCCCTAATGTATGTGATCTTGGAAAATGCCCCGGACGTGAGGGAAGCGGCACGGAGAATTGAAGAAGgtgagatggtattttattgatttttatagaaattgaaaaatatctttttgggtttac
+3fr: S··Q··S··S··E··V··F··F··D··N··C··Q··K··L··L··D··K··F··N··Y··P··W··E··L··M··P··L··M··Y··V··I··L··E··N··A··P··D··V··R··E··A··A··R··R··I··E··E··

----////----

1118126 EXON F

0.76 atttaccacttcacgatctAGgtcaatgcgttgtgaatgag

DNA: gcatttgtgtgaggattaaactttgacaaatgattgctaaacaacatttaccacttcacgatctagGTCAATGCGTTGTGAATGAGTATACTCGAATGCATAATCTCAATATGTATGATGGAGCCGAATTGAGGGGCTCCACCCGGCAATGCGGATGATAAtttactgccagcagtttcatatgttagtaaaaaaaaaacaaacgtTCATCAATCAACAttgagataatatgcaaatgtt
+3fr: G··Q··C··V··V··N··E··Y··T··R··M··H··N··L··N··M··Y··D··G··A··E··L··R··G··S··T··R··Q··C··G··*··*·

DNA: ggcactcgtacacacctaagttcaaactaagatatatcgtaacttaacccaagaaatgcgtatttgaaagaaagatcaaccccgttaagcttacacaaaactcttttattgattagatttactaattaattgtagatgtacataatatgagtatggccacttcttttcattctttttatatatttagtaattaatatcgtttaaaacttcttggagtgcgcatttttaattgcttaaaaa

DNA: gaatatttgtggataattattattttaaatgattatttatgattgttcgcggtagtggctattggtgttaaagtgatTCTTCAATCAACAtttTCTTCAATCAACAttTCTTCAATCAACACCTTCAATCAACAaatctgcaatggaaacaaaaaaatcaagctcaaaaatcacaaaacatatcttccataatttttgactcaattataatgatgtttttttggcgggaaatgccaaaag

1118868

0.70 tcacaatgtgagaaa

DNA: gacccacttaaatcagactctcaagagagaagaaaatcaatcaaccatttggatttagccacaatcttaacagtacatccattcacaatgtgagaaatgggcagcgtttcatcaaattgacaacaatatagatgtggatgccagaagattgcaacagcgttaaaaaaagtggaacaacagcaaaaaaagtgcaaggagaaaacacattaatgttcaaaggcgaatggtaagaagttgatgt

----////----

1141155 EXON M

0.57 atcaggatgtttattttgcagctgcgagtataataaaatgt

DNA: tatcggccactgaagagcccgcgagtcattggttaatcatgattaccggatattacaatattattagttaagtaatcaggatgtttattttgcagCTGCGAGTATAATAAAATGTCGATATGGACATGGACTCCCAATCCCAGCACACCAGTACTACACATACCTATCTACGGCGGCAGCGAGAGTGCAGACGTCGATATCCATATCCTTCGTACTACTACACATACTGGGCAGCCGGTA
+2fr: · A··A··S··I··I··K··C··R··Y··G··H··G··L··P··I··P··A··H··Q··Y··Y··T··Y··L··S··T··A··A··A··E··C··R··R··R··Y··P··Y··P··S··Y··Y··Y··T··Y··W··A··A··G··S

1141498

DNA: TACAAGTCCCTTCCTATATAACTTCTATAACGAGACAGTACTGTCGCCGCATTTGCACAGCAAGTACCTCAATTCGGAAAGTACTCCTGCGGTACGGAATATATCGGAATCTCCGTCAGCCTATTCATCTCTGCCACTTCCTGCCGCTCTACAAATTACTCGTCTGATACCAACGAATAGCTTATCTGGTGCATCGACGTAGcgatgtatactctatgtgattcaactaatagtgattgt
+2fr: ··T··S··P··F··L··Y··N··F··Y··N··E··T··V··L··S··P··H··L··H··S··K··Y··L··N··S··E··S··T··P··A··V··R··N··I··S··E··S··P··S··A··Y··S··S··L··P··L··P··A··A··L··Q··I··T··R··L··I··P··T··N··S··L··S··G··A··S··T··*·

Figure S21. Manually-curated *P. papatasi doublesex* gene model. Exonic sequences are indicated by black cases; black upper cases indicate coding sequences. Intronic sequences are indicated by gray lower cases. Start and stop codons are highlighted in green and red, respectively. In yellow boxes, putative TRA/TRA-2 binding sites. Exon start/end positions in the scaffold/s are indicated. Intron boundaries were predicted using transcripts vs genome alignments and confirmed by *de novo* prediction with Berkeley BDGP Splice Site Prediction Tool with default parameters (http://www.fruitfly.org/seq_tools/splice.html); prediction scores are indicated in red.

L. longipalpis dsx gene model

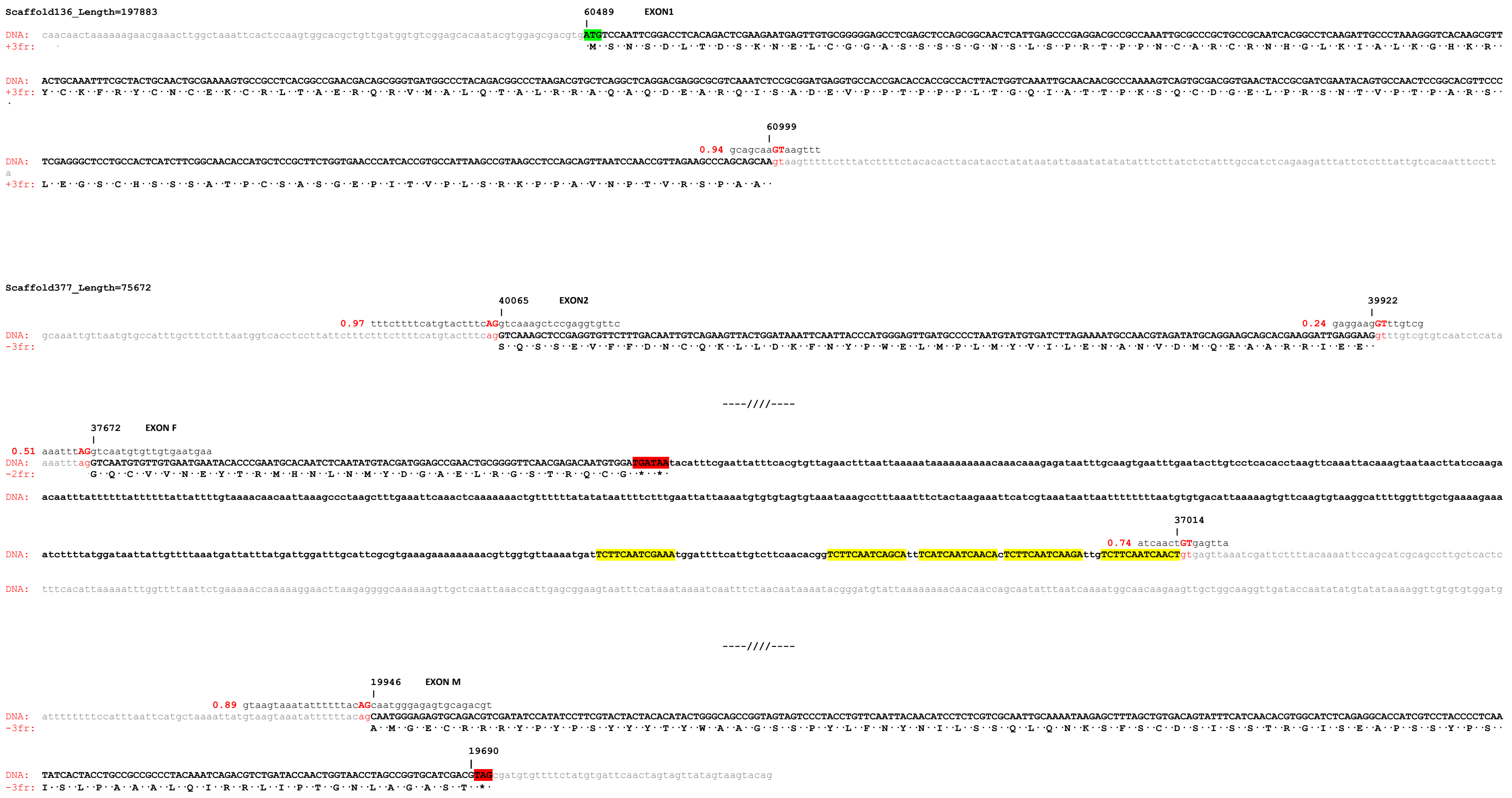

Figure S22. Manually-curated *L. longipalpis doublesex* gene model. Exonic sequences are indicated by black cases; black upper cases indicate coding sequences. Intronic sequences are indicated by gray lower cases. Start and stop codons are highlighted in green and red, respectively. In yellow boxes, putative TRA/TRA-2 binding sites. Exon start/end positions in the scaffolds are indicated. Intron boundaries were predicted using transcripts vs genome alignments and confirmed by *de novo* prediction with Berkeley BDGP Splice Site Prediction Tool with default parameters (http://www.fruitfly.org/seq_tools/splice.html); prediction scores are indicated in red.

P. papatasi fru gene model

A)

Scaffold43920_Length=20651

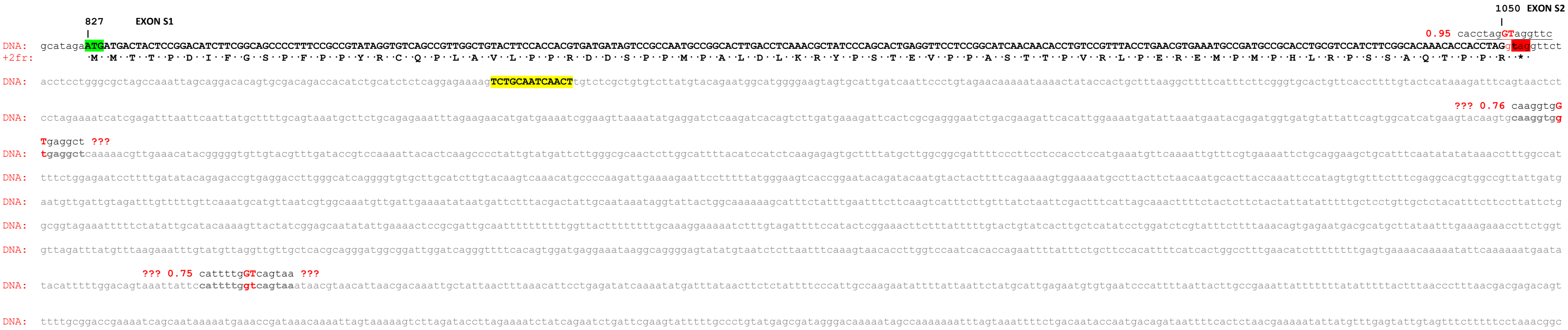

Figure S23A. Manually-curated *P. papatasi fruitless* gene model. The genomic region regulated by sex-specific alternative splicing, which includes the two sex-specific exons S1 and S2, is reported. Exonic sequences are indicated by black cases. Black upper cases indicate coding sequences. Intronic sequences are indicated by gray lower cases. Start codons are highlighted in green. In yellow boxes, putative TRA/TRA-2 binding sites. Exon start/end positions in the scaffolds are indicated. Intron boundaries were predicted using transcripts vs genome alignments and confirmed by *de novo* prediction with Berkeley BDGP Splice Site Prediction Tool with default parameters (http://www.fruitfly.org/seq_tools/splice.html); prediction scores are indicated in red. Red question marks indicate putative predicted female-specific donor splice sites. The absence of the expected TRA/TRA-2 binding site cluster in the proximity of female-specific donor splicing site in Scaffold 43920 indicates a possible incorrect assembly of this genomic region.

P. papatasi fru gene model

B)

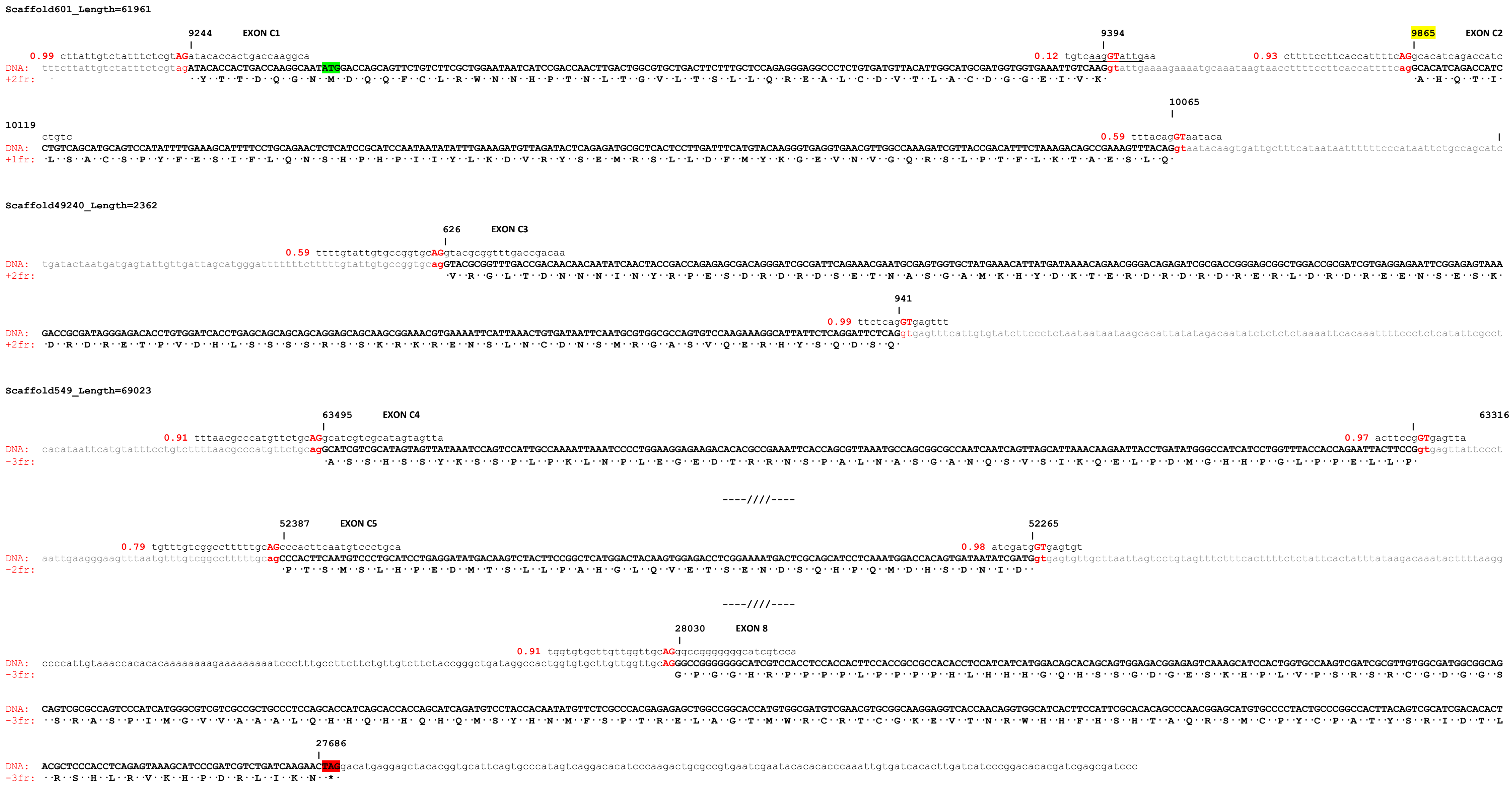

Figure S23B. Manually-curated *P. papatasi fruitless* gene model. The genomic region which includes common exons 1-5 and the terminal exon, encoding for the Zinc Finger domain of type C, is reported. Exonic sequences are indicated by black cases. Black upper cases indicate coding sequences. Intronic sequences are indicated by gray lower cases. Start and stop codons are highlighted in green and red, respectively. In yellow boxes, putative TRA/TRA-2 binding sites. Exon start/end positions in the scaffolds are indicated. Intron boundaries were predicted using transcripts vs genome alignments and confirmed by *de novo* prediction with Berkeley BDGP Splice Site Prediction Tool with default parameters (http://www.fruitfly.org/seq_tools/splice.html); prediction scores are indicated in red. Red question marks indicate putative predicted female-specific donor splice sites. The absence of the expected TRA/TRA-2 binding site cluster in the proximity of female-specific donor splicing site in Scaffold 43920 indicates a possible incorrect assembly of this genomic region.

L. longipalpis fru partial gene model

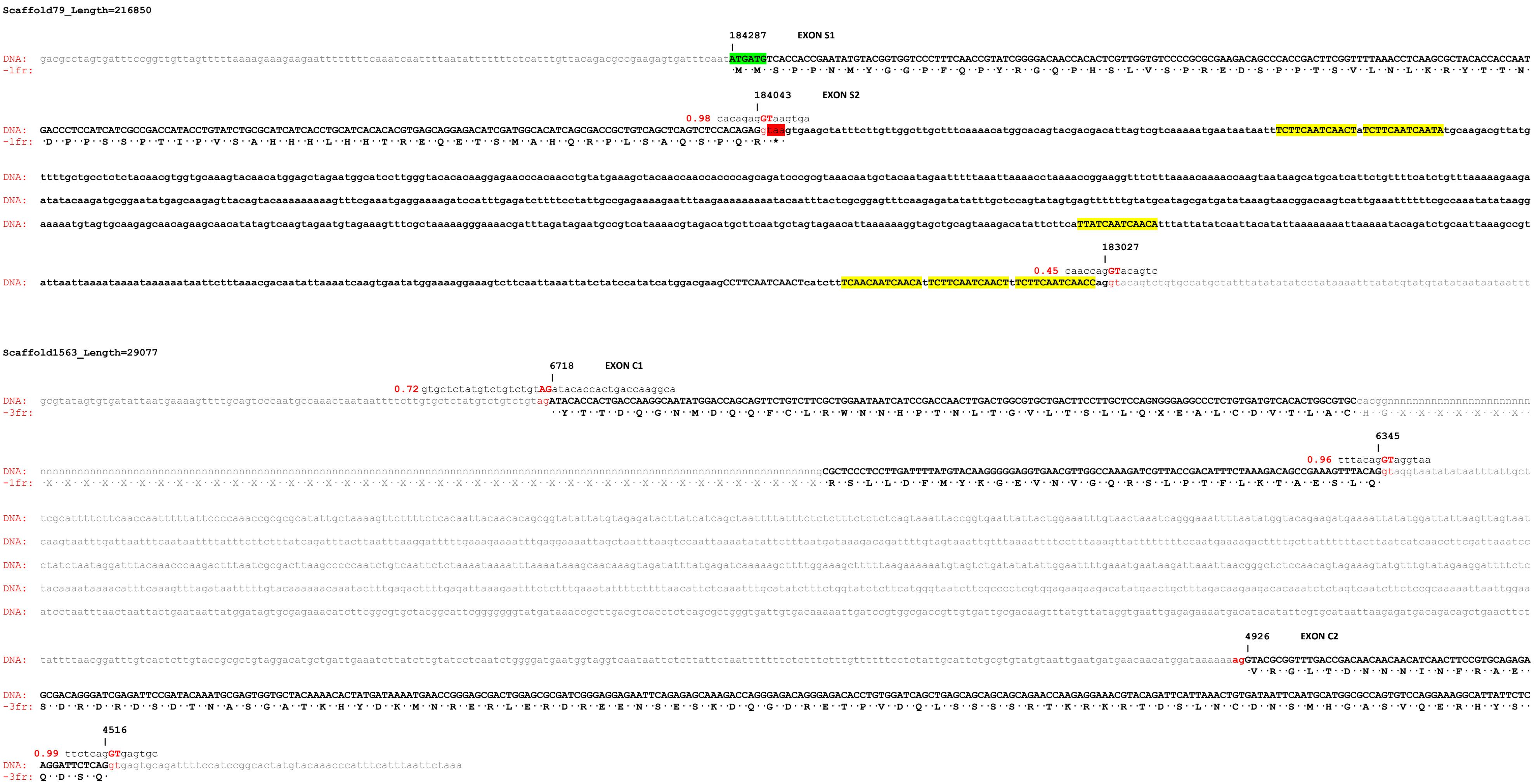

Figure S24. Manually-curated *L. longipalpis fruitless* partial gene model. The genomic region regulated by sex-specific alternative splicing is included. Exonic sequences are indicated by black cases. Black upper cases indicate coding sequences. Intronic sequences are indicated by gray lower cases. Start and stop codons are highlighted in green and red, respectively. In yellow boxes, putative TRA/TRA-2 binding sites. Exon start/end positions in the scaffolds are indicated. Intron boundaries were predicted using transcripts vs genome alignment and confirmed by *de novo* prediction with Berkeley BDGP Splice Site Prediction Tool with default parameters (http://www.fruitfly.org/seq_tools/splice.html); prediction scores are indicated in red.

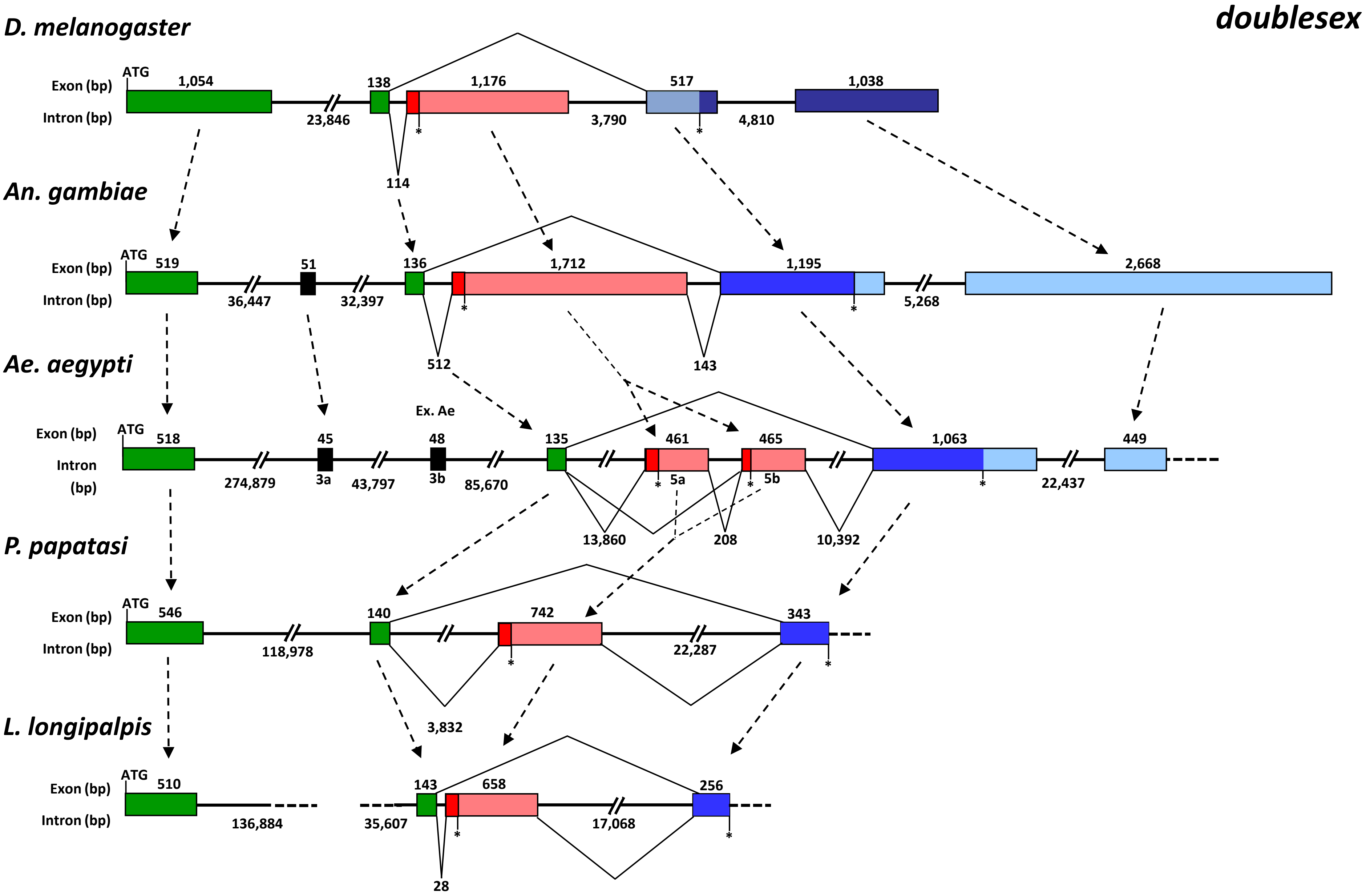

Figure S25. Comparison of genomic structures of diptera *dsx* genes. Green boxes represent the OD1 and OD2 domains encoding common exons. Black boxes represent exons encoding protein regions conserved in mosquitoes but not in fruit flies and sand flies. Alternative male-specific and female-specific exons are represented as blue boxes and pink boxes, respectively. Asterisks indicate the position of stop codons. In *Drosophila*, the *dsx* gene encompasses a 45 Kb-long region on chromosome 3R and is organized into 6 exons and 5 introns, with three common exons followed by a female-specific and two male-specific exons. DmDSXF translation initiates at the ATG within exon 2 and terminates within the female-specific exon 4, while in the case of DmDSXM, translation begins at the same ATG and terminates within the first male-specific exon 5.

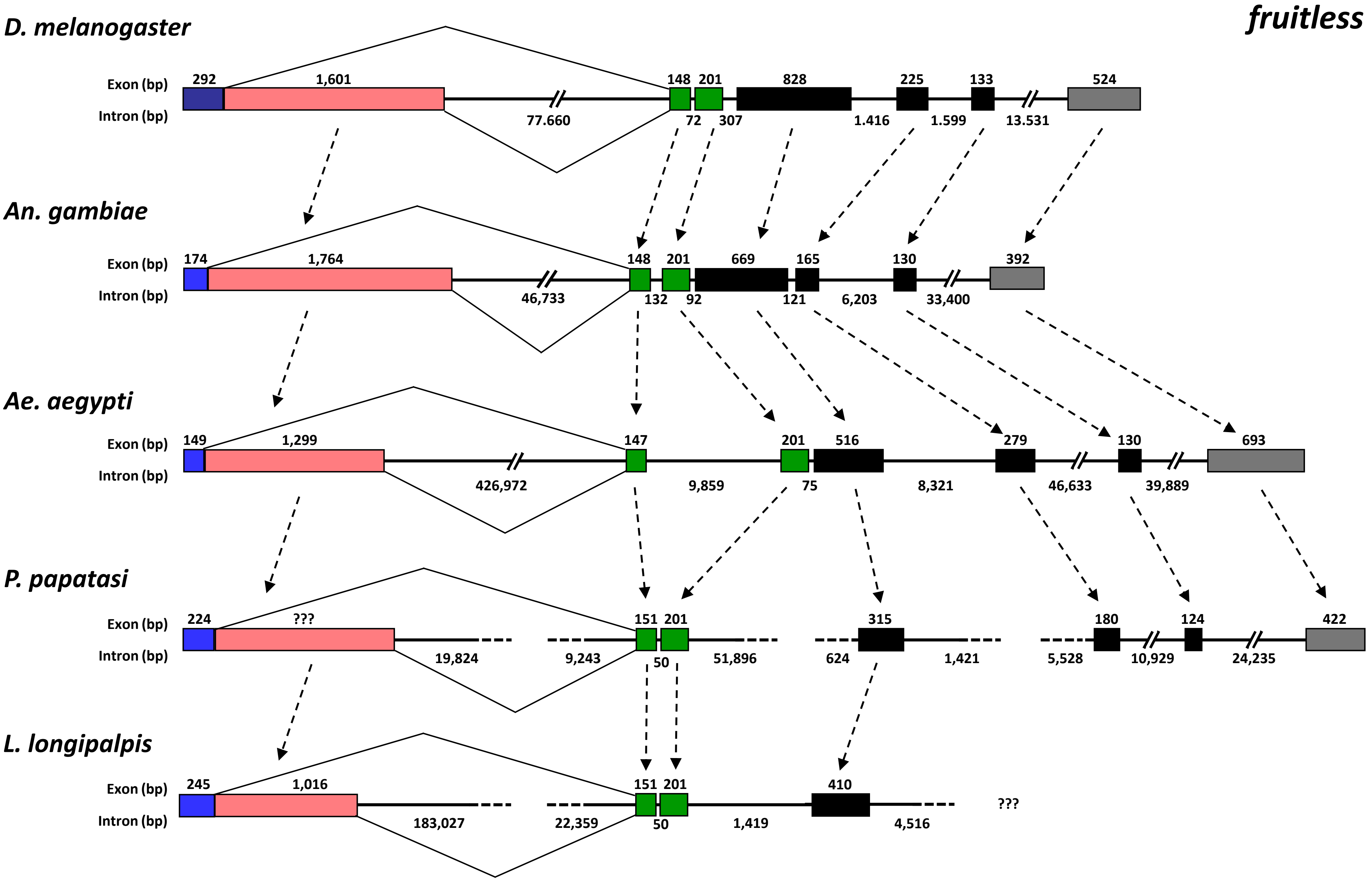

Figure S26. Comparison of genomic structures of diptera *fru* genes. Fru S1 common (but encoding the male-specific N-terminus) and S2 female-specific exons are represented as blue and pink boxes, respectively. Green boxes represent the non-sex-specific C1 and C2 exons, encoding the BTB domain. Black boxes represent the non-sex-specific C3-C5 exons encoding the Connector region of FRU proteins. The terminal grey boxes represent the ZnF-C domain encoding exons. The *Drosophila fru* gene encompasses a 98 Kb-long region and is organized in 7 exons and 6 introns with 6 common exons preceded by the sex-specific regulated region, with a common exon and a female-specific exon.
