# Supplementary material for: Identification of sex determination genes and their evolution in Phlebotominae sand flies (Diptera, Nematocera)
Source: BMC Genomics. 2019 Jun 25;20:522. doi: 10.1186/s12864-019-5898-4 (PMC6593557; doi:10.1186/s12864-019-5898-4)
Supplement: Supplementary file 12 — Figure S28. Manually-curated P. bergeroti fruitless partial gene model. Figure S29. Manually-curated P. duboscqi fruitless partial gene model. Figure S30. Manually-curated P. bergeroti doublesex partial gene model. Figure S31. Manually-curated P. duboscqi doublesex partial gene model. (PDF 142 kb) [file 12864_2019_5898_MOESM12_ESM.pdf]

P. bergeroti fru partial gene model

Contig_256375_Lenght=2168

```
                                                                                                                                                                                                                                                                                                                       1948
                                                                                                                                                                                                                                                                                                                       |
DNA:  gctgaacactaacaatagttcttcacaagaaaaaattaccaaataaattatcaaattgtaaatttctgtatttctttaaaagatttgaactgtataaaagatactaattttttttgaaaaatagcagtaaaatatcatcatacaattatatttttaactaaaatttgcatgtgctatctctctatttgcagagcttgagcagagtaagtgatcgcatagaATGATGACTACTCCGGACA
-2fr:                                                                                                                                                                                                                                                                                                                   ·M··M··T··T··P··D··
                                                                                                                                                                                                                                                                                       1724
                                                                                                                                                                                                                                                                                       |
                                                                                                                                                                                                                                                                          0.96 cacctagGTaggttc
DNA:  TCTTCGGCAGCCCGTTTCCGCCGTATAGGTGTCAGCCGTTGCCTGTACTTCCACCACGTGATGATAGTCCGCCAATGCCGGCGCTTGACCTTAAACGCTATCCCAGCACTGAGGTCCCACCGGCATCAACAACACCTGTCCGTTTATCTGAACGTGAAATGCCGGTGCCGCACCTGCGTCCATCTTCAGCACAAACACCACCTAGgtaggttctacctcctgggcgctagccaaattag
-2fr: I··F··G··S··P··F··P··P··Y··R··C··Q··P··L··P··V··L··P··P··R··D··D··S··P··P··M··P··A··L··D··L··K··R··Y··P··S··T··E··V··P··P··A··S··T··T··P··V··R··L··S··E··R··E··M··P··V··P··H··L··R··P··S··S··A··Q··T··P··P··R··*·

DNA:  caggacacagtgcgacagatcacatctgcatctctcaggagaaaagTCTGCAATCAACTtgtctcgctgtgtcttatgtacagaatggtatggggaagtagtgcgctcccctgccgtatttcctcatccactgtgaaaaccctgatccaacccgccatccctgcgtgagcaacaacctaacatacaaatttcttaaacataaatctaacaccagaaggtttctttcaaattataagcat

DNA:  gcgtcattctcactgtttaaaagaaatacgagatccaggatatgagcaagtgatacagtacaaaaataaagaagtttccgagtatggaaaatctagaaagatttttccttagcaaaaaaaagtaaccaaaaaaaaaaatttgcaatcgcggagttttcaatatattgctccgatagtaacttttgtatgcaatatagaaaaatttctaccgccagaataaggaagaaatgtagagcaaca

DNA:  ggagcaaaaatataatagtagaagagtagaaaagtttgctaaagaaagtcgaattagataaacaagaaatgacttgaagaaattcaaatagaaatgctttttttgccagtaataactatttattgcaatagtcgtaaagaatcattataTTTTCAATCAACAtttgccacaattaacatgcatttgaacaaaaatataTCTACAATCAACATtcatcaataacggccacgtgcctcgaaa

                    953
                    |
          1.00 gaatttgGTaagtgc
DNA:  gaaacacactatggaatttggtaagtgcattgttagaagtaaggcattttccacttttctgaaaagtagtacattatatctgtattccggtgacttcccataaaaaggaattcttttcaatcttcgggcatgtttgacttgtacaagatgcaagcacacccctgatgcccaaggtcctcacagtccctgtatatcaaaaggattctccagaaaatggccaaaggtttatatatattgaa

DNA:  atgcagctttctgtagaattttcacgaaacaattttgaacattcatgggggaggaggaagggaaaatcgccgccaagcataaaagcactctcttgagatggatgtaaaatgccaagagttgcgcccaagaatcatacaataggggcttgagtgtaattttggacggtatcaaacgtacaacacccctgtatgtttcaacgtttttgagcctcaccaccttgcacttgtacttcatgatg

DNA:  ccactgaataatacatcaccatctcgtattcatttaatatcattttccaatgtgaatcttcgtcagattccctcgcgagtgaatctttcatcaagactgtgatcttgagatcctcctattttaacttccgatttcatcatgttcttctaaatttctctgtagaagcatttactgctaaagcataattgaattaaatctcgatgattttctagcagagttatactgaaatttttatgagt

DNA:  acaaaaggtgaacagttcacccaaagaaatgaaaagtcttaaagcagtggtagttttattttttgtgctacagggaattgatcagtttatgtaatctcttaatttcaaattaacacatttgtcattcgtcacacactagaattttatttctgcttccacatattaatcactgtctttgaatgaaagactgcctttgagtgaaaataaaaatatgcagttttggtcagtaggggaaagtac

DNA:  tctctctccgaacgttc
```

Contig_16679_Lenght=1684

```
DNA:  tatatatatatatatatatatatatgttctatgccttatataatatatactctatatacattatatagtaaaatcaataattttttaatacattcgatcttttacaaatctttttaaataaaattaaaagaggctttgacacatctattaaacaaaatacttttattcatatttttctattcatttgggagactttttttttgagacaatcttcctgggtcaccctgttcataaaactttg

DNA:  agtgttaatgaaggctttttttgcctcttccatgtgtgttctccgacgcgaatagaaaaagacagatatgacagtatgttgcaaaattttagaataaatttattttctagatgctcatttttttcgcaaacacctcgcccatgtataagcgccaaaaggtttttgcaggtggaactggacatggtcaataaaaaattagtctttctaccttgatacaatccgtgtgcaaaactttccatt

                                                                                                                           1105
                                                                                                                           |
                                                                                                 0.99 cttattgtctatttctcgtAGatacaccactgaccaaggca
DNA:  aggtttttttttttggtaaaatagtcgaggagtgatattaatgaaaagttttgcactttcaatgacaaactaataaatttttcttattgtctatttctcgtagATACACCACTGACCAAGGCAATATGGACCAGCAGTTCTGTCTTCGCTGGAATAATCATCCGACCAACTTGACTGGCGTGCTGACTTCTTTGCTCCAGAGGGAGGCCCTCTGTGATGTTACATTGGCATGCGATGGTGG
-1fr:                                                                                                                          ··Y··T··T··D··Q··G··N··M··D··Q··Q··F··C··L··R··W··N··N··H··P··T··N··L··T··G··V··L··T··S··L··L··Q··R··E··A··L··C··D··V··T··L··A··C··D··G··G
                954
                |
  0.89 tgtcaagGTatttat
DNA:  TGAAATTGTCAAGgtatttattttattacactgcaattactgaaataattatcttttttttttgttgataagttcatccctcttgagtgtcataaaacatgttcgcgaatttcttttagtatagtgcaaaatgtaggaaaaacaaagaatagtaattatatagtacccgagtcttaatgttttcgtcaccggttcctaatttaattatactattatactattagtatcaaaaaattacc
-1fr: ··E··I··V··K·

                                                                                                                                                                                                                                                                                                 0.98 ctttt
DNA:  ttaatagaagaaaagacagacagatattgacgtcaatcgatatgtggagctagaaaccttgggcaacgcgttaaaaagattcaaaaactcgctttttgctccatctcactctcgcctacatcaccatcaatgtcttggactatctcactttgtcttgtcttgtgagacacaatattctaagatgtagaaaaaaaaattcttggtgtcattgaaaagaaaatgcaaatacgtaacctttt

                      450                                                                                                                                                                                                                                                      250
                      |                                                                                                                                                                                                                                                        |
      ccttcaccattttcAGgcacatcagaccatcctgtc                                                                                                                                                                                                                         0.72 tttacagGTaataca
DNA:  ccttcaccattttcagGCACATCAGACCATCCTGTCAGCATGCAGTCCATATTTTGAAAGCATTTTCCTGCAGAACTCTCATCCGCATCCAATAATATATTTGAAAGATGTTAGATACTCAGAGATGCGCTCACTCCTTGATTTCATGTACAAGGGTGAGGTGAACGTTGGCCAAAGATCGTTACCGACATTTCTAAAGACAGCCGAAAGTTTACAGgtaatacaagtgattgctttca
-1fr:                 ·A··H··Q··T··I··L··S··A··C··S··P··Y··F··E··S··I··F··L··Q··N··S··H··P··H··P··I··I··Y··L··K··D··V··R··Y··S··E··M··R··S··L··L··D··F··M··Y··K··G··E··V··N··V··G··Q··R··S··L··P··T··F··L··K··T··A··E··S··L··Q·

DNA:  taataatttttcccataattctgccagcatcttaaattgttgtttagtgtcagaattcagcataattctcacaattacatcccagcggtagcacatataattccaattaaagcttctttcctgagaattggcagagaaatgatcaagaattagtattagagttgaagatttttttctctagatggaattttcaaatacaattgcgaaaattcctttcgtaattttgacataaaaatcta

DNA:  aaattaaaaa
```

Figure S28. Manually-curated *P. bergeroti fruitless* partial gene model. The genomic region regulated by sex-specific alternative splicing is included. Exonic sequences are indicated by black cases. Black upper cases indicate coding sequences. Intronic sequences are indicated by gray lower cases. Start and stop codons are highlighted in green and red, respectively. In yellow boxes, putative TRA/TRA-2 binding sites. Exon start/end positions in the scaffolds are indicated. Intron boundaries were predicted using transcripts vs genome alignment and confirmed by *de novo* prediction with Berkeley BDGP Splice Site Prediction Tool with default parameters (http://www.fruitfly.org/seq_tools/splice.html); prediction scores are indicated in red.

P. duboscqi fru partial gene model

Contig_1876045_Length=2257

DNA: gtgaaagattacacactataatagtgtaaagttaaattaaatttcagatgaattgttcttttgcgttattttgtattaaaaaccatttgaattgatagattttcgcttatatctctgtaaaaatatataaaatttgaaattttctatgttatttttagccctaaatatatcaaaataaaaatttctgtatttcgttaaaatattcaaaattttgttaaaaaggaacaggaaaattcca

1935

DNA: taatattataatattttaactaaaacttgtgtttcttatctgtctatttacagagcttgagaagagtaagtgaccgcatagaATGATGACTACTCCGGACATCTTCGGCAGCCCCTTTCCACCGTATAGGGGTCAGCCGTTGGCTGTACTTCCACCACGTGATGACAGTCCGCCAACGCCGGCACTTGACCTCAAACGCTATGCCAACACTGAGGTCCCTCCGGCATCAACAACACCT
-2fr: ·M··M··T··T··P··D··I··F··G··S··P··F··P··P··Y··R··G··Q··P··L··A··V··L··P··P··R··D··D··S··P··P··T··P··A··L··D··L··K··R··Y··A··N··T··E··V··P··P··A··S··T··T··P·

1685
0.96 cacctagGTaggttc

DNA: GTCGCACCACACCTCCGTTTACCGCTGCATCATGCACATGAACGTGAAATGCCGATACCGCACCAGCGTCCATCTTCGGCACAAACACCACCTAGgtaggttctacctcctgggcgctagccaaattagcaggacacagtgcgacagatcacatctgcatctctcaggagaaaagTCTGCAATCAACAtgtctcgctgtgtcttatgtacagaatgggtatggggaagtagtgcgctcc
-2fr: ·V··A··P··H··L··R··L··P··L··H··H··A··H··E··R··E··M··P··I··P··H··Q··R··P··S··S··A··Q··T··P··P··R··*·

DNA: cctgccttatttcctcatccactgtgaaaaccctgatccaacccgtcatccctgcgtgagcaacaacctaacatacaaatttcttaaccataaatctaacaccaaaggtttctttcaaattataagcatgcgtcattctcactgtttaaaagagatacgagatccaggatatgagcaagtgatacagtacaaaaaaagaagtttccgagtatggaaaatctagaaagatttttccttt

DNA: gcaaaaaaaagtaaccgaaaaaaattgcaatcgcggagtttttaatatattgctccgatagtaacttttgtatgcaatatagaaaaatttctaccgccagaataaggaagaaatgtagagcaacaggagcaaaaatataatagtagaagagtagaaaagtttgctaaagaaagtcgaattagataaacaagaaatgacttgaagaaattcaaatagaaatgctttttgccaataatac

920
1.00 gaatttgGTaagtgc

DNA: ctatttattgcaatagtcgtaaagaatcattataTTTTCAATCAACAtttgccacgattaacatgcatttgaacaaaaataaaTCTACAATCAACAttcatcaataacggccacgtgcctcgaaagaaacaaactggaatttggtaagtgcattgttagaagtaaggcattttccactgttttctgaaaagtgtagtacattgtatctgtattccggtgacttcccataaaaaggaatt

DNA: cttttcaatcttcgggcatgtttgacttgtgcaagatgcaagcacacccctgatgcccaaggtccttacggtctctgtatatcaaaaggattctccagaaaatggccaaaggtttatatatattgaaatgcagctttctgcagaattttcacgaaacaattttgaacattcatggtggaggaggagaaaatcgccgccaagcataaaagcactctcttgagatggatgtaaaatgccaa

DNA: gagttgcgcccaagaatcatacaatagggggcctgagtgtaattttggacggtatcaaacgtacaacacccccgtagtatgtttcaacgtttttgagcctcaccaccttgcacttgtacttcatgatgccactgaataatacattaccatctcgtattcatttaatatcattttccaatgtgaatcttcgtcagattccctcgcgagtgaatctttcatcaagactgtgatcttgagatc

DNA: ctcctattttaacttccgatttcatcatgttcttctaaatttctctgcagaagcatttactgcaaaagcataattgaattaaatctcgatgattttctaggagagttactgaaatttttatgagtacaaaaggtgaacagtacacccaatgaaatgaaaagtctttaagcactgatagttttatttttgttctacagggaattgatcaatttatgtaatctcttaattacaaagtaaca

DNA: ccttggtttaattccatcagaattttatttctccttccacattttcaccactgcttttaaatagaagtctgtctttgataaataataaataataataataatgctg

Contig_84267_Lenght=896

DNA: aaaaatatgtatcttaactatttgcaatgaaagatagtttctgggatttaattgatcaataaaaatatattcacttaaacaatattataaggctgtgtaatatatttatatagcatcagtcttttaatctttaacatattcgatcttttacaaatatttttaactgaaattgaaaaaggctttggcaaattaatttaacaaaatactttttttcgtactctcctattcatttgggagact

DNA: ttttttgagacaatcttcctgggtcaccctgttcataaaactttgagtgttaatgaaggtttttttgcctcttccatgtgtgttctccgacgcgaatagaaaaagacagatatgacagtatgttgcaaaatttagaataaatttattttctagatgctcatttttttcgcaaacacctcgcccatgtataagcgccaaaaggtttttgcaggtggaactggacatggtcaataaaaaat

624
0.99 cttattgtctatttctcgtAGatacaccactgaccaaggca

DNA: tagtctttctaccttgatacaatccgtgtgcaaaactttccattaggtgctttttttttggtaaaatagtcgaggagtgatattaatgaaaagttttgcactttcaatgacaaactaataaattttcttattgtctatttctcgtagATACACCACTGACCAAGGCAATATGGACCAGCAGTTCTGTCTTCGCTGGAATAATCATCCGACCAACTTGACTGGCGTGCTGACTTCTTTGCT
-1fr: ··Y··T··T··D··Q··G··N··M··D··Q··Q··F··C··L··R··W··N··N··H··P··T··N··L··T··G··V··L··T··S··L··L

121
0.89 tgtcaagGTatttat

DNA: CCAGAGGGAGGCCCTCTGTGATGTTACATTGGCATGCGATGGTGGTGAAATTGTCAAGgtatttattttattaaattgaaatacttaagtttttttaaataagttcatccctcttgagtgccataaaacatgttcgcaaatttcttctagcgttgtgcaaaatgtagaaaaacgaaaaaa
-1fr: ··Q··R··E··A··L··C··D··V··T··L··A··C··D··G··G··E··I··V··K·

Contig_34439_Length=735

DNA: tctccggttcctaatttaattatactattatactattagtatcaaaaaattaccttaatagaagaaaagacagacagatattgacgtcaatcgatatgtggagctagaaaccttgggcagcgcgttaaaaagattcaaaaacttgctttttgctccatctcactctcgcctacatcaccatcaatgtcttggactatctcactttgtcttgtcttgggagacacaatattctaagatgt

310
0.98 cttttccttcaccattttcAGgcacatcagaccatcctgtc

DNA: attaaaaaaaaattcttggtgtcattgagaagaaaatgcaaataagtaaccttttccttcaccattttcagGCACATCAGACCATCCTGTCAGCATGCAGTCCATATTTTGAAAGCATTTTCCTGCAGAACTCTCATCCGCATCCAATAATATATTTGAAAGATGTTAGATACTCAGAGATGCGCTCACTCCTTGATTTCATGTACAAGGGTGAGGTGAACGTTGGCCAAAGATCGTTA
+3fr: ·A··H··Q··T··I··L··S··A··C··S··P··Y··F··E··S··I··F··L··Q··N··S··H··P··H··P··I··I··Y··L··K··D··V··R··Y··S··E··M··R··S··L··L··D··F··M··Y··K··G··E··V··N··V··G··Q··R··S··L·

511
0.72 tttacagGTtaataca

DNA: CCGACATTTCTAAAGACAGCCGAAAGTTTACAGgtaatacaagtgaatagctttcataatttttcccataattctgccagcatctaaattgttgtttagtgtaagacattcagcataattctcacaattacatcccagcggtagcacatataagtccaattaaagcttctttgctgagaattggcagagaaatgatcgaaaattagtattagagttgaaatgaattattaatagatttt
+3fr: ·P··T··F··L··K··T··A··E··S··L··Q·

DNA: ttttctctagatggaatt

Figure S29. Manually-curated *P. duboscqi fruitless* partial gene model. The genomic region regulated by sex-specific alternative splicing is included. Exonic sequences are indicated by black cases. Black upper cases indicate coding sequences. Intronic sequences are indicated by gray lower cases. Start and stop codons are highlighted in green and red, respectively. In yellow boxes, putative TRA/TRA-2 binding sites. Exon start/end positions in the scaffolds are indicated. Intron boundaries were predicted using transcripts vs genome alignment and confirmed by *de novo* prediction with Berkeley BDGP Splice Site Prediction Tool with default parameters (http://www.fruitfly.org/seq_tools/splice.html); prediction scores are indicated in red.

P. bergeroti dsx partial gene model

Contig_33004_Length=2707

```
DNA:  gcccgaatttgagagagtctactgtatggtgagcattttactgtcaatttgattcttctctgaactgaatttagaacttctttttccgagtgtatgtcgactcgattttgcaaataaaggggagcttctggacatatatctctctcactttcatcgatacggtttttcttttcgcaattattgcacacactttcatgttccatgtgcgccactgcaacaccaacaagcaaatggtcagac

DNA:  ggtgggtagtaacattgcatatgctatatgttgatgcatagcagacatttagttctgaacttgcacacactttcatgttccatgtgcgccactgcaacaccaacaagcaaatggtcagacatgtgtgagtaatgattgcaaatatatattcaagtgaatgacgttgagcggcgaaatatcttggcattgacgcgatttagccgcgaagagagaactcaagcactcgtcagtgtgtcgata

DNA:  cgtatgggattgtattatatgatctgtatgcactatcgcaaccagaccgaaagacccatggttatgtattcagttgcaaccgtcctccgttcgtgtcacactggctttcaaattcctgtcgctacaattcgatatcctcagtttgtttgacaccaccatcggcaacagttgcactgaatcccaaaaaataagattccgccgagattttcctaatcagcttgtgcatttcgataaatcgtg

DNA:  taccacgcggcatagcgctgcatggtgctttgtgctcgagtaataatttctttttcttctttggacaatttactgcttgtgtgaatattgagaaggtgaaattgagagattgttagttgagaataattgtttcacatgaaaatattctcctgtgaaatttctgcaaaagacataggagtgagacacaaaaaaagagggaagtttcattccttgtacttttaataaaatactgactgaatt

DNA:  ccaatgttactaccccatggtgttgattgaatgaagtgcaaagcagaaaatgttgattaattgcctgtaataccacaaacgttcattggtgaatcttcacgtaatgatcatcaaaaataaactcaagtcctaaagcattatatcggcataacgtctgaatctgacgaagaaagaaaaaaaaaactataaatactccattagctcatttgattgtgaaggtgaatttattaaatgtcttct

DNA:  ttgattgcaacattggcgcactttcttacccaagagagcttctcaactgagaagaacctggggtagtcggttgcatgtgcactaaacttatatgccaacaattgtatacatttttgtgacttgttgcttgctacgaagtacataaatacagtggaagtggcaattatataggcacttttatcatacagctctcaccacgaaaagaaaaaacaatatagcagaggaggtagacctcttagc

DNA:  ttggtactgaagaaaaaagtttgagtttgtgtagaattgcaagctttcgacctgctactaccaagactatttatttttttattttaattgtgcaaactttgtgcagaaaacgtgccatctgcgagagactcacagaggttaaatctcttccgtgatccacatcagtgacaattaatgattctaacttagaagattatcatactgatttctatataagatcaatccatttaaactgttcaag

DNA:  acataccttaggttttgatcaaatacccatcgctagcaaagtgttggggtcttcaatcatatcgcatttgaaatccctaccctcgagacattggccagaaagtcttatcgttaattgacgatattttcttgagtcatcacagagttatgccagaagacaatctgtataagaaggaatgagagaaagaagtggcaaaaagtgatgaatgtttgcctttggttggggtaacttcggcagatgt

                                                                                                                                                                                                                                                                         2091
                                                                                                                                                                                                                                                                         |
DNA:  ctacagacctgtaaaattgcaataagagacaatttaaagacattaaaacatctgacaagtgattattgcaaattcagtaacattgtgtgcaaaaaagtgactaaattctctgtataatctagactaagtttcagtcttcggtcaaaggttcaattaagtgggacgctgttgATGGTGTCGGAGCACAATACGTGGAGCGACGTGATGTCCAATTCGGACCTCACAGACTCGAAAAACGAG
+1fr:                                                                                                                                                                                                                                                                     M  V  S  E  H  N  T  W  S  D  V  M  S  N  S  D  L  T  D  S  K  N  E

DNA:  TTGTGCGGAGGAGCCTCGAGCTCCAGCGGCAACTCATTGAGCCCGAGAACGCCACCAAATTGCGCCCGCTGCCGCAATCACGGCCTCAAGATAGCCCTTAAAGGTCACAAACGCTACTGCAAGTACCGTTACTGCAACTGCGAAAAGTGTCGTTTAACGGCCGAACGTCAGCGGGTGATGGCCTTGCAGACGGCCCTAAGACGTGCTCAGGCTCAGGACGAGGCACGTCAGATCTCCGCG
+1fr:  L  C  G  G  A  S  S  S  S  G  N  S  L  S  P  R  T  P  P  N  C  A  R  C  R  N  H  G  L  K  I  A  L  K  G  H  K  R  Y  C  K  Y  R  Y  C  N  C  E  K  C  R  L  T  A  E  R  Q  R  V  M  A  L  Q  T  A  L  R  R  A  Q  A  Q  D  E  A  R  Q  I  S  A

                                                                                                                                                                                                                                                                                                              2578
                                                                                                                                                                                                                                                                                                              |
                                                                                                                                                                                                                                                                                                    1.00 gctgcaaGT
DNA:  GATGAGGTGCCACCGACGCCACCGCCAGCCCCGTCCATTTGCACCCTTGCCACTCCTAAGAGTCAGTGCGATAGCGAACCACCGGCACCAATTACTGTGCCCACACCGGCACGTTCCCTAGAGGGTTCCTGCCACTCATCATCGGCCACACCCTGTTCTGCTTCCGGAGAACCCATCTCCGTGCCATCCAGCCGCAAATTGGCCCCAGTCAATCCAACCATCAGAAATCCAGCTGCAAgt
+1fr:  D  E  V  P  P  T  P  P  P  A  P  S  I  C  T  L  A  T  P  K  S  Q  C  D  S  E  P  P  A  P  I  T  V  P  T  P  A  R  S  L  E  G  S  C  H  S  S  S  A  T  P  C  S  A  S  G  E  P  I  S  V  P  S  S  R  K  L  A  P  V  N  P  T  I  R  N  P  A  A

      aagtac
DNA:  aagtaccattcaaattatccttacaacatataaggtactctctacaacagctaccctacttatggcttcaaacattaaaaaagaaatatgcttaatcctaagagttcaggaatttttgtaaaacttt
```

Contig_197637_Length=1025

```
DNA:  tccttctattcttagtcattctatttccttatcaattagtcaacagacatagtaaaagtacaggtgaagtgtcttgaggtgtgtagtttttttattcgcaacttgatttcatcaaaaggcactcaagatgatggtgaaggaaaaagtctttgccaatcaatgacttcaacaattatttcaatttaatattatttaccctttttggtatcgggaaagatgcttttaaaagatatcatcgtgt

                                                                                                                                                                                                                                                                                                          551
                                                                                                                                                                                                                                                                                                          |
                                                                                                                                                                                                                                                                                 0.93 ttcctcttgtcacgctttcAGgtcaaa
DNA:  ggcagagtccgtggagatcacagattcgcaataattttatattgacgcaccttttgatatataatatttgattttaatggaagctaatgatagactgcaacacgcaaatatttcccttttactattcattaaataccatgccataaaaaaagagccaacacttaaactagattctgtaattgatggaatattttcttgattttaatgtacaactcttcctcttgtcacgctttcagGTCAAA
-3fr:                                                                                                                                                                                                                                                                                                          G  Q

                                                                                                                                                                                   407
                                                                                                                                                                                   |
      gctccgaagtcttc                                                                                                                                                  0.99 gaagaagGTaagatg
DNA:  GCTCCGAAGTCTTCTTTGACAATTGCCAGAAATTGCTGGATAAATTCAATTACCCTTGGGAACTGATGCCCCTAATGTATGTGATCTTGGAAAATGCCAATGTTGACGTGAGGGAAGCGGCACGGAGAATTGAAGAAGgtaagatggattttttattgatttgatttaaaaaaaaatgaaaaatatcttttcggttttacgtgaaacccattgcattgtgattcctgaaatgattttgagatgaaa
-3fr: S  S  E  V  F  F  D  N  C  Q  K  L  L  D  K  F  N  Y  P  W  E  L  M  P  L  M  Y  V  I  L  E  N  A  N  V  D  V  R  E  A  A  R  R  I  E  E

DNA:  gacataaatttggcatgagatcctactagataatccaatcaaatcaaatttattaagaagaaggttccgttgttaagaactagtttagctagattccatcgactaataaaattgaagaaaataaatcttcctgttgtagagacaattacttttatgtcaaattagatcaatttcaatttagtagtctataggcagtacaagacttttaactattaaaattttttatttactgatgaagaac

DNA:  ttaatatgcaagttttatgtgaatagaacattagttaaattacgtctaagaaattgcataatggt
```

Contig_120456_Length=776

```
                                                                                                                                                                          148
                                                                                                                                                                          |
                                                                                                                                                  0.24 atttaccacttcacgatctAGgtcaatgcgttgtgaatgag
DNA:  aagttacttaagacacctaaggcaatgccctttctaattcgattcagtttcaaatgttactcattttcaatgggatttgagtgcattttagcgaggattaaactttgacaaatgatttctaaacaacatttaccacttcacgatctagGTCAATGCGTTGTGAATGAGTATACTCGAATGCATAATCTCAATATGTATGATGGAGCCGAATTAAGGGGCTCCACCCGGCAATGCGGATGA
-1fr:                                                                                                                                                                       G  Q  C  V  V  N  E  Y  T  R  M  H  N  L  N  M  Y  D  G  A  E  L  R  G  S  T  R  Q  C  G  *

DNA:  TAAttactgccagcagtttcatatgttagtaaaagaaaaaaacgtTCATCAATCAACAttgagagaatatgctaatgttggcactcgtacacacctaagttcaaactaagatatatggtaacttaacccaagaaacgcgtatttgaaagaaagatcaaccccgttaagcttacacaaaactcttctattgattagatttactaattaattgtagatgtacataatatgagtacggccac
-1fr:  *

DNA:  ttcttttcattctttttatatatttagtaattaatatcgtttaaaacttcttggagtgcgcatttttatttgcttaaaaagaatatttgtggataattattgttttaaatgattatttatgattgttcgcggtagtggctattggtgttaaagtgatTCTTCAATCAACAtttTCTTCAATCAACAttTCTTCAATCAACACTTTCAATCAACAaatctgcaatggaaataaaaaatcaa

DNA:  actcaaaaatcacaaaacatatctttcataatttttgactcaattataatgatgtt
```

Figure S30. Manually-curated *P. bergeroti doublesex* partial gene model. The genomic region regulated by sex-specific alternative splicing is included. Exonic sequences are indicated by black cases. Black upper cases indicate coding sequences. Intronic sequences are indicated by gray lower cases. Start and stop codons are highlighted in green and red, respectively. In yellow boxes, putative TRA/TRA-2 binding sites. Exon start/end positions in the scaffolds are indicated. Intron boundaries were predicted using transcripts vs genome alignment and confirmed by *de novo* prediction with Berkeley BDGP Splice Site Prediction Tool with default parameters (http://www.fruitfly.org/seq_tools/splice.html); prediction scores are indicated in red.

P. duboscqi dsx partial gene model

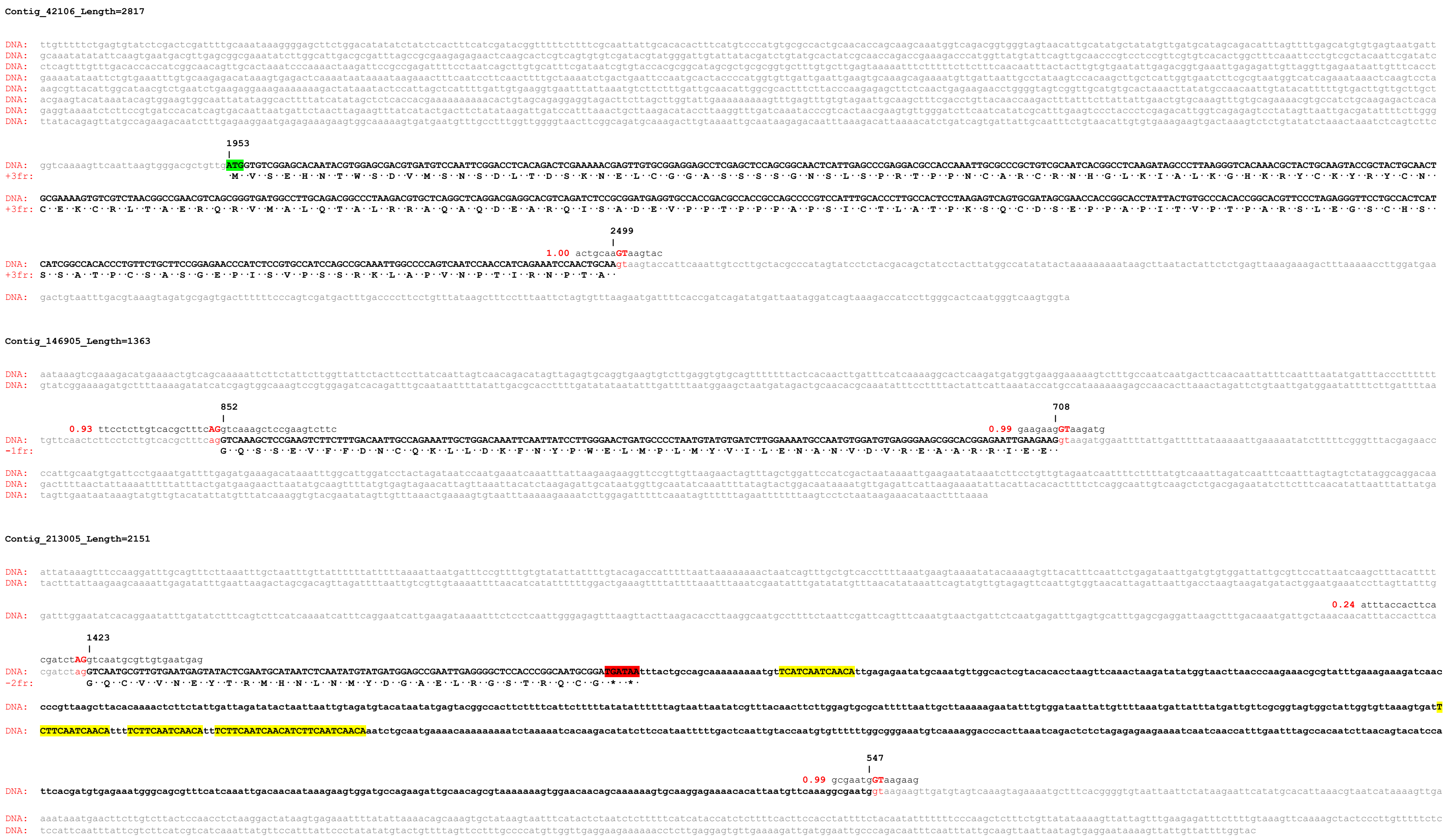

Figure S31. Manually-curated *P. duboscqi doublesex* partial gene model. The genomic region regulated by sex-specific alternative splicing is included. Exonic sequences are indicated by black cases. Black upper cases indicate coding sequences. Intronic sequences are indicated by gray lower cases. Start and stop codons are highlighted in green and red, respectively. In yellow boxes, putative TRA/TRA-2 binding sites. Exon start/end positions in the scaffolds are indicated. Intron boundaries were predicted using transcripts vs genome alignment and confirmed by *de novo* prediction with Berkeley BDGP Splice Site Prediction Tool with default parameters (http://www.fruitfly.org/seq_tools/splice.html); prediction scores are indicated in red.
