# Supplementary material for: Identification of sex determination genes and their evolution in Phlebotominae sand flies (Diptera, Nematocera)
Source: BMC Genomics. 2019 Jun 25;20:522. doi: 10.1186/s12864-019-5898-4 (PMC6593557; doi:10.1186/s12864-019-5898-4)
Supplement: Supplementary file 15 — Supplementary Methods. (PDF 345 kb) [file 12864_2019_5898_MOESM15_ESM.pdf]

Supplementary Methods

Table of contents

1) Cloning of *P. perniciosus fru* transcripts
2) Genome assembly of *P. bergeroti* and *P. duboscqi*
3) Manual curation of *tra*, *tra-2*, *dsx* and *fru* gene models in Phlebotominae
4) *De novo* transcriptome assembly of sand fly species
5) List of primers utilized in this study
6) Nucleotide sequences of the sex determining genes identified by *in silico* approach
7) Supplementary Methods References

1) Cloning of *P. perniciosus fru* transcripts

The TBLASTN search against the perniBASE dataset using the *A. aegypti* FRU proteins as query (Tab. S1) led us to identify two partially *in silico* assembled transcripts encoding for partial male-specific and non-sex-specific Fruitless (FRU) protein isoforms, both containing a well conserved BTB domain. We further searched within the perniBASE dataset by TBLASTN using as queries the *D. melanogaster* FRU Zinc-Finger terminal domain of type A, B and C (ZnF-A, ZnF-B and ZnF-C) (Salvemini et al. 2013) and we identified two transcripts, c23972.g4.i1 and c25556.g5.i1, encoding for partial proteins with a well conserved ZnF-A or ZnF-C domains, respectively. These transcripts could represent uncomplete assembled *Ppefru* transcripts. We validated this hypothesis by gene specific RT-PCR analysis, using reverse primers located in the two ZnF putative transcripts c23972.g4.i1 and c25556.g5.i1 (PpeFruZnfA-, PpeFruZnfAinner-, PpeFruZnfC-, PpeFruZnfCinner-) and a forward primer located in the *P. perniciosus* male-specific *fru* transcript c23888.g1.i4 (PpFruM+). We successfully amplified four full length cDNAs (*PpefruMA*, 2249 bp; *PpefruFA*, 3026 bp; *PpefruMC*, 1915 bp; *PpefruFC*, 2692 bp) with complete FRU ORFs in male and females of *P. perniciosus*. The four cDNAs were cloned and sequenced as described in Methods.

2) Genome assembly of *P. bergeroti* and *P. duboscqi*

We produced a draft genome assembly of the sand fly species *P. bergeroti* and *P. duboscqi* using the MINIA assembler (Salikhov et al., 2013) and the available Illumina genomic reads at SRA NCBI Archive (https://www.ncbi.nlm.nih.gov/sra) (*P. bergeroti*: SRR1973671 and SRR1973788; *P. duboscqi*: SRR1973598 and SRR1996565) with the following command:

```
minia -in species_name.fasta -kmer-size 31 -abundance-min 3 -out species_name_assembly_k31_m3 -nb-cores 23
```

We obtained the two following genome assemblies:

Assembly statistics	*P. duboscqi*	*P. bergeroti*
Total number of assembled sequences	2240724	1741498
Total length of sequence	351743961 bp	359027491 bp
Total GC count	123284946 bp	127899439 bp
GC %	35.05	35.62
Average sequence length	156 bp	206 bp
Minimum length	63 bp	63 bp
Maximum length	32976 bp	29034 bp
N50 stats >=	190 bp	365 bp
Number of Sequences longer than 10000	45	22
Number of Sequences longer than 5000	469	680
Number of Sequences longer than 3000	2802	4894
Number of Sequences longer than 2000	11514	18082
Number of Sequences longer than 1000	51627	64808

3) Manual curation of *tra*, *tra-2*, *dsx* and *fru* gene models in Phlebotominae

The *P. perniciosus* TRA, TRA-2 DSX and FRUM protein sequences and the *P. papatasi* and *L. longipalpis* orthologous predicted proteins PpaDSXF (PPATMP010078-RA and PPATMP010079-RA), LloDSXF (LLOTMP001672-RA and LLOTMP005763-RA), LloDSXM (LLOTMP005763-RA), PpaFRUM (PPATMP009581-RA) and LloFRUM (LLOTMP008996-RA) were utilized as queries to perform a TBLASTN search against the genomic data set available at VectorBase (https://www.vectorbase.org/) or the newly assembled *P. bergeroti* and *P. duboscqi* draft genomes to identify the approximate position of intron/exon boundaries. Exact intron boundaries were localized by using transcripts vs genome nucleotide alignments and were confirmed by *de novo* prediction with the Berkeley BDGP Splice Site Prediction Tool using the default parameters (http://www.fruitfly.org/seq_tools/splice.html). This approach led us to reconstruct complete *tra* gene models in *P. perniciosus*, *P. papatasi*, *P. bergeroti* and *P. duboscqi* (Fig. S10-S13), partial *tra-2* gene models in *P. perniciosus*, *P. papatasi*, *P. bergeroti* and *P. duboscqi* (Fig. S17-S19), the complete *dsx* gene model in both *P. papatasi* and *L. longipalpis* species (Fig. S21-S22), the complete *fru* gene model in *P. papatasi* (Fig. S23A-B) and a partial *fru* gene model in *L. longipalpis* (Fig. S24).

4) *De novo* transcriptome assembly of sand fly species.

We produced a *de novo* transcriptome assembly of the Old World sand fly species *L. longipalpis*, *L. umbratilis* and *L. neivai* using the Trinity assembler (Grabherr et al., 2011; Haas et al., 2014) and the following Illumina RNA-seq reads from SRA NCBI Archive (https://www.ncbi.nlm.nih.gov/sra):

Species	Run	life stage	sex
L. longipalpis	SRR535756	adult	female
L. longipalpis	SRR535757	adult	female
L. longipalpis	SRR535758	fourth instar larvae	<not provided>
L. longipalpis	SRR535759	fourth instar larvae	<not provided>
L. longipalpis	SRR535760	adult	female
L. longipalpis	SRR535761	adult	female
L. longipalpis	SRR535762	adult	female
L. longipalpis	SRR535763	adult	female
L. longipalpis	SRR535764	adult	female
L. longipalpis	SRR535765	adult	female
L. longipalpis	SRR535766	adult	female
L. longipalpis	SRR535767	adult	female
L. longipalpis	SRR535768	adult	female
L. longipalpis	SRR535769	adult	female
L. longipalpis	SRR535770	adult	female
L. longipalpis	SRR535771	adult	female
L. longipalpis	SRR535772	adult	female
L. longipalpis	SRR535773	adult	female
L. longipalpis	SRR535774	adult	female
L. longipalpis	SRR535775	adult	female
L. longipalpis	SRR535776	adult	female
L. longipalpis	SRR535777	adult	female
L. longipalpis	SRR535778	adult	female
L. longipalpis	SRR535779	fourth instar larvae	<not provided>
L. umbratilis	SRR952151	adult	<not provided>
L. neivai	SRR5134059	adult	<not provided>
L. neivai	SRR5134060	adult	<not provided>

L. neivai	RR5134061	adult	<not provided>

Downloaded reads were combined for each species in single or paired fastq files, were quality filtered and adaptor trimmed with Trimmomatic software (Trimmomatic-0.32) (Bolger et al., 2014) and assembled using the following Trinity commands:

L. longipalpis:

```
Trinity --seqType fq --max_memory 180G --left longipalpis_1.fq --right longipalpis_2.fq --CPU 23 --jaccard_clip --normalize_reads --output longipalpis_trinity
```

L. umbratilis:

```
Trinity --seqType fq --max_memory 180G --left umbratilis_1.fq --right umbratilis_2.fq --CPU 23 --jaccard_clip --normalize_reads --output umbratilis_trinity
```

L. neivai:

```
Trinity --seqType fq --max_memory 180G --single nyssomyia.fq --CPU 23 --normalize_reads --output neivai_trinity
```

We obtained the following transcriptome assemblies:

Assembly statistics	*L. longipalpis*	*L. umbratilis*	*L. neivai*
Total number of assembled transcripts	236076	176358	116069
Total number of Trinity genes	166427	148912	75760
Total assembled bases	198156135	81656931	68762545
GC %	40.37	41.84	38.52
Average sequence length	839 bp	463 bp	592 bp
Median transcript length	483 bp	304 bp	317 bp
N50 stats >=	1368 bp	520 bp	921 bp

5) List of primers utilized in this study

Primer name	Sequence (5'--3')	Tm (°C)
PpeSOD+	AGGAGGCGTCGTTCGTGAAT	56
PpeSOD-	CCTTGCCATGAGGATTGTAG	60
Ppdsx5'utrCommon+	GTGTGTGGATTCTACTAAGG	54
PpdsxCommon+	TCAGACGTGCTCAGGCTC	58
PpdsxF-	CGGCTCCGTCATACATATTA	54
Ppdsx3'utrF-	CTTCGCTGCTTGGGATAAGT	60
PpdsxM-	TACTCGTTGGTATCAGGCG	56
PpFruM+	CGACAAAAGACACTCTATTTC	54
PpefruC-	ATGGTCTGATGAGCCTTGAC	58
PpFruF+	GGATATGAGCAAGAGATACAG	52
PpFruC-nested	GTCGGATGGTTATTCCAGCG	58
PpeFruZnfA-	GTCTGACTTTGATGACTAATTGG	60
PpeFruZnfAinner-	CAATCTCGCCATTCTGGTTC	58
PpeFruZnfC-	CAACATTTTCTCCACAGAGG	56
PpeFruZnfCinner-	ACAATCTTCGTTGGTACTGTC	58
ppetra_F2inner	AATGGCGACAAAGAGGGAGG	58
Ppetra_Rinner	TTCTCCCTCAAGCGATCCCT	62
PpeTraRace3+	CAGAGGATTCAGATAAGGAG	56
PpeTraRace3Nested+	GAGTCGTAGTCGGAGTCTC	54
Tra-5’Race_outer	CGTCCGTTTGTGTTGAGAG	54
Tra-5’Race_inner	TATGACTTTCCTCCCTGTGG	58
PpeTra5'utr	GATCCAATTGTGCAATTGTC	50
PpeTraStop3utr-	CTATTCAGTCAATTTTCAATGG	58
PpeTra-2_F1-6-5	CACCAGATTATGCACTTTTGC	58
PpeTra-2_R	CCTATGATACATTTACAAGTAAGG	64
PapTraF	CATGTTCCATCTACATCAC	52
PapTraR	AAGGATCGTACCAGTCATAC	54
LutzTraF	ACACAAAAGGACGGGTAATG	56
LutzTraR	TTTGTTCGTCTCCGGCGAG	60

6) Nucleotide sequences of the sex determining genes identified by *in silico* approach

```
>Pbetra_CDS [Phlebotomus bergeroti] P. bergeroti transformer CDS
ATGATTAAAAAACCTAGCTCCAGGACGCACAAGGAAAAATATGAGGGAAAATACAAAACAAATGGCGGAAGATCAGCATCGAA
GAGGGAGGCAGAGAGACATCCATCAACACATGAAGACACCATCAAAAAATCCCATCATCATCGTGATGAATTAGGAAAGAAAG
ATGAAAGGAAGTCTTATGAGGATGATTTTCCCAAACTTGGGCATGTTCCATCTACATCACGGGATACCCGGGACGGCTTTCAA
CACAAAAGAACGGGTGATGGCAGGGAATCCTCTAAGCATCGAAGAGGTGCTGCCAAGAGAGATTCTTCAAATTCTTCAGAGAC
TGACAGTGAATCCCTCAAATCTTCGAGGAAAAGGCCAGAATCTCGCCGGAGACGATCAAAAAGTCGTAGCAGAAGTCTCGATG
ACTCGTACAGGAGACGTCAGACCTCCACGAGTCGTCGATCTTCACCACGACGCCGGCCAAAGTCTGTTTCTCCACCGCGGGAG
AAAGTGCCCTACTATGCAGATGCCCAGCGTGAGAGGGATCGCCTGAGAGAGAAGTATGGAAGTGATGGGAGGATGAGGAGGAG
GAGCAGCAGCCGCGAGAGACGCAGAAGACGCTCTAGAGATAGAAGATCCAAGACTCCAGTCCGAGAAACCACAAAAAATTGTCA
CAGTTCCTGTTCCTGTTCCCGTCTACCCATCAATTTATCCAGATGGTTCCATGTATGACTGGTACGATCCCTCTTGGCAAACA
GGGCTCTTTCCACAGGGTGTAATGCCTCCAAGACACATTCCACGCCCAATGCGACCTCATGCCCCATTCCCTACTTCACCCTT
CATGGTGGATCCTTTTCATCGGCCAATGAGGCCAGGAATGCCACCTCCAAGACCCTTTCGACCTCCCCAAGGATTCCCTCCTC
CCCGATTCCACAGACCCCATTGA
>Pdutra_CDS [Phlebotomus duboscqi] P. duboscqi transformer CDS
ATGATTAAAAAACCTAATTCCAAGACGCATAAGGAAAAATATGAGGGAAAATACAAAACAAATGGCGGAAGATTGGCATCGAA
GCGAGAAGCAGAGAGACATCCATCAACACATGAAGACACCATTAAAAAATCCCATCATCATCGTGATGAATTAGGGAAGAAAG
ATGAAAGGAAGGCTTATGAAGAAGAATTTCCTAAATTTGGGCATGTTCCATCTACATCACGGGATTCCCGGGACGGCTTTCAA
CACAAAAGGACGGGTGATGGTAGGGAATCCTCTAAGCACCGAAGAGGTGCTGCCAAGAGAGATTCTTCAAATTCTTCAGAGAC
TGACGATGAATCCCTCAAATCTTCGAGGAAAAGGTCAGAATCTCGCCGGAGACGATCTAAAAGTCGTAGCCGAAGTCTGGATG
ACTCGTACAGGAGACGTCAAACCTCCACGAATCGTCGATATTCGCCACGGCGCCGGCCAAAGTCTGTTTCTCCTCCTCGTGAG
AAAGTTCCCTACTATGCAGATGCCCAACGCGAGAGGGATCGCCTGAGGGAGAAATATGGAAGTGATGGGAGGGCTAGGAGAAG
GAGCAGCAGTCGCGAGAGACGCAGAAGGCGTTCTAGAGAAAGGAGATCCAAGACTCCAGTCCGAGAAACCACAAAAAATTGTTA
CAGTTCCTGTTCCTGTTCCTGTCTACCCGTCAATATATCCGGATGGTTCCATGTATGACTGGTACGATCCTTCTTGGCAAGCA
GGACTCTTTTCTCAGGGTGTCATGCCTCCAAGACACATTCCACGCCCAATGCGTCCTCATGCCCCATTCCCTGCCTCACCCTT
CATGGTGGATCCTTTCCATCGGCCAATGAGACCAGGAATGCCACCTCCAAGACCCTTCCGACCTCCCCAAGGATTCCCTCCTC
CCCGATTCCACAGACCCCATTGA
>Ppatra-2_CDS [Phlebotomus papatasi] P. papatasi transformer-2 CDS
ATGAGTACACGTACCTACTATAAGAACTCAATGTCAAGAAGTCCCAGCGAGGAACGACACTATATCTCTCGAGCTGGTTCATC
GCGAGAGAATGGCGACTACAGGAAGTCCCGCAAATCGTCTTCCCGCAGTCGAGCCCGCTACAGATCTCGCTCACGTTCACGGT
CTCGTTCTGAGAAGCGCTACAGAAGCAGGTCACCGTCCCGCCACGATAGGAAGCGCTCCTATTCCAGGAGCCCCAATTCCTCT
CGTCGCAGACACGTAGCAAGTCGAGTCAATTCACAGGATCATCCCCCAAAGTCCAGATGTCTAGGAGTCTTTGGACTCAGTGA
TGCAACAACTGAAGACCAGATTTATCAAATTTTCTCCAAATTTGGAACAATACACCGTACTCAAATAATTATGGATGCAATGA
CGGGATGTTCACGGGGCTTCTGTTTTGTCTACTTTGCCAATGCTGATGATGCCAAAGTGGCAAAGGATAATTGCTCAGGAATG
GAACTCGATGGGAGACGTATTCGAGTGGATTATTCCATTACTCAGAGACCTCATACTCCAACTCCAGGAGTCTACATGGGACA
ACCAACGGGCAGAGGACGAGAGAATCGAGATCGTAGTCGCAGAAATGAGTAA
>Lumtra-2_par_CDS [Lutzomyia umbratilis] L. umbratilis transformer-2 partial CDS
AGAAATCTCTTCCCACAGTCATCAGATAGCTCCGGGTACGACAGGAGGCACTCCAGGACAAAGAAGAAGCGTGAAAATGGGCA
CCACAAGAAATCATCAAAATCCCGAAGGCGCCATCGATCAAGGTCTCACTCAAAGCGACGACACTACTCACGATCACCCACTA
GGACCACCCGGAAGCGCCACATCTCACGCAGCCCAGTCCCAACATACAAGAAATATGAACAACCGGAAGAGGGAAGATGCCTG
GGTGTCTTTGGATTGAGCATGCACACAACAGAGAAGCACGTGCATGAGATCTTTTCCAAATTCGGACCCATTGAACGAACGCA
AATCATTGTGGATTCAAAGACTGGTCGATCTCGGGGATACTGCTTCGTTTATTTTGAAAATACTGAAGATGCAAAGGTAGCCA
AGGATCAATGTACTGGGATTGAGATTGATGATCGTCGCATTCGTGTGGATTATTCCCTCACAGCAAGACCCCATACACCTACA
CCTGGAATTTATATGGGTAAGGCTAGTCAACGGTGTGGGGACTCATCAACATCACGAAGCAACAGATACGAGGATACTTCTTA
CGAGAAAACTCGTAACAGATCACCATCCCCATACAAACGTAGGCGCCGAAGTCGATCTCGTTCACACTCACCAAGAGTTCGCC
GATATCATTAA
>Lnetra-2_par_CDS [Nyssomia neivai] N. neivai transformer-2 partial CDS
AGAAATCTCTTCCCACAGTCATCAGATAGTTCCGGGGACGACAGGAGGTACTCCAGGACAAAGAAGAAGCGTGAGAATGGGCA
CCACAAGAAATCATCAAAATCCCGAAGGCGCCATCGATCAAGGTCTCACTCAAAGCGACGACACTACTCACGATCTCCCACTA
GGACCATCCGGAAGCGGCACATCTCACGCAGCCCAGTCCCATCATACAAGAAATATGAACAACCGGAAGAGGGTAGATGTCTG
GGTGTCTTTGGGTTGAGCATGCACACAACAGAGAAGCATGTTCATGAGATCTTCGCCAAATTCGGACCCATTGAACGAACGCA
AATTATCGTGGATTCAAAGACTGGTCGTTCTCGGGGATACTGCTTTGTTTATTTTAAAAATACTGAAGATGCAAAGGTAGCCA
AGGATCAATGTACTGGGATTGAGATTGATGATCGTCGTATTCGTGTGGATTATTCCCTAACAGCAAGACCCCATACACCGACA
CCTGGAGTTTATATGGGCAAGGCTAGTCAACGGTGTGGGGATCGTGGGGACTCATCAACATCACGAAGCAACAGATACGAGGA
TTCTTCTTACGAGAAGACTCGCAACAGATCACCATCCCCTTATAAACGCAGGCGCCGAAGTCGATCCCGCTCATACTCACCAA
GAGTTCGCCGATATCACTAA
>PpadsxF_CDS [Phlebotomus papatasi] P. papatasi doublesexF CDS
```

ATGGTGTCGGAGCACAATACGTGGAGCGACGTGATGTCCAATTCGGACCTCACAGACTCGAAAAACGAGTTGTGCGGAGGAGC
CTCGAGCTCCAGCGGCAACTCATTGAGCCCGAGGACGCCACCAAATTGCGCCCGCTGCCGCAATCACGGCCTCAAGATAGCCC
TTAAGGGTCACAAACGCTACTGCAAGTACCGTTACTGCAACTGCGAAAAGTGTCGTCTAACGGCCGAACGTCAGCGGGTGATG
GCCTTGCAGACGGCCCTAAGACGTGCTCAGGCTCAGGACGAGGCACGTCAGATCTCCGCGGATGAGGTGCCACCGACGCCACC
GCCAGCCCCGTCCATTTGCACCCTTGCCACTCCTAAGAGTCAGTGCGATAGCGAACCACCGGCACCCATTACGGTGCCCACAC
CGGCACGTTCCCTAGAGGGTTCCTGCCACTCATCATCGGCCACACCCTGTTCTGCTTCCGGAGAACCCATCTCCGTGCCATCC
AGCCGCAAATTGGCCCCAGTCAATCCAACCATCAGAAATCCAACTGCAAGTCAAAGCTCCGAAGTCTTCTTTGACAATTGCCA
GAAATTGCTGGACAAATTCAATTATCCTTGGGAACTGATGCCCCTAATGTATGTGATCTTGGAAAATGCCCCGGACGTGAGGG
AAGCGGCACGGAGAATTGAAGAAGGTCAATGCGTTGTGAATGAGTATACTCGAATGCATAATCTCAATATGTATGATGGAGCC
GAATTGAGGGGCTCCACCCGGCAATGCGGATGATAA
>PdudsxF_CDS [Phlebotomus duboscqi] P. duboscqi doublesexF CDS
ATGGTGTCGGAGCACAATACGTGGAGCGACGTGATGTCCAATTCGGACCTCACAGACTCGAAAAACGAGTTGTGCGGAGGAGC
CTCGAGCTCCAGCGGCAACTCATTGAGCCCGAGGACGCCACCAAATTGCGCCCGCTGTCGCAATCACGGCCTCAAGATAGCCC
TTAAGGGTCACAAACGCTACTGCAAGTACCGCTACTGCAACTGCGAAAAGTGTCGTCTAACGGCCGAACGTCAGCGGGTGATG
GCCTTGCAGACGGCCCTAAGACGTGCTCAGGCTCAGGACGAGGCACGTCAGATCTCCGCGGATGAGGTGCCACCGACGCCACC
GCCAGCCCCGTCCATTTGCACCCTTGCCACTCCTAAGAGTCAGTGCGATAGCGAACCACCGGCACCTATTACTGTGCCCACAC
CGGCACGTTCCCTAGAGGGTTCCTGCCACTCATCATCGGCCACACCCTGTTCTGCTTCCGGAGAACCCATCTCCGTGCCATCC
AGCCGCAAATTGGCCCCAGTCAATCCAACCATCAGAAATCCAACTGCAAGTCAAAGCTCCGAAGTCTTCTTTGACAATTGCCA
GAAATTGCTGGACAAATTCAATTATCCTTGGGAACTGATGCCCCTAATGTATGTGATCTTGGAAAATGCCAATGTGGATGTGA
GGGAAGCGGCACGGAGAATTGAAGAAGGTCAATGCGTTGTGAATGAGTATACTCGAATGCATAATCTCAATATGTATGATGGA
GCCGAATTGAGGGGCTCCACCCGGCAATGCGGATGATAA
>PbedsxF_CDS [Phlebotomus bergeroti] P. bergeroti doublesexF CDS
ATGGTGTCGGAGCACAATACGTGGAGCGACGTGATGTCCAATTCGGACCTCACAGACTCGAAAAACGAGTTGTGCGGAGGAGC
CTCGAGCTCCAGCGGCAACTCATTGAGCCCGAGAACGCCACCAAATTGCGCCCGCTGCCGCAATCACGGCCTCAAGATAGCCC
TTAAAGGTCACAAACGCTACTGCAAGTACCGTTACTGCAACTGCGAAAAGTGTCGTTTAACGGCCGAACGTCAGCGGGTGATG
GCCTTGCAGACGGCCCTAAGACGTGCTCAGGCTCAGGACGAGGCACGTCAGATCTCCGCGGATGAGGTGCCACCGACGCCACC
GCCAGCCCCGTCCATTTGCACCCTTGCCACTCCTAAGAGTCAGTGCGATAGCGAACCACCGGCACCAATTACTGTGCCCACAC
CGGCACGTTCCCTAGAGGGTTCCTGCCACTCATCATCGGCCACACCCTGTTCTGCTTCCGGAGAACCCATCTCCGTGCCATCC
AGCCGCAAATTGGCCCCAGTCAATCCAACCATCAGAAATCCAGCTGCAAGTCAAAGCTCCGAAGTCTTCTTTGACAATTGCCA
GAAATTGCTGGATAAATTCAATTACCCTTGGGAACTGATGCCCCTAATGTATGTGATCTTGGAAAATGCCAATGTTGACGTGA
GGGAAGCGGCACGGAGAATTGAAGAAGGTCAATGCGTTGTGAATGAGTATACTCGAATGCATAATCTCAATATGTATGATGGA
GCCGAATTAAGGGGCTCCACCCGGCAATGCGGATGATAA
>LlodsxF_CDS [Lutzomyia longipalpis] L. longipalpis doublesexF CDS
ATGTCCAATTCGGACCTCACAGACTCGAAGAATGAGTTGTGCGGGGGAGCCTCGAGCTCCAGCGGCAACTCATTGAGCCCGAG
GACGCCGCCAAATTGCGCCCGCTGCCGCAATCACGGCCTCAAGATTGCCCTAAAGGGTCACAAGCGTTACTGCAAATTTCGCT
ACTGCAACTGCGAAAAGTGCCGCCTCACGGCCGAACGACAGCGGGTGATGGCCCTACAGACGGCCCTAAGACGTGCTCAGGCT
CAGGACGAGGCGCGTCAAATCTCCGCGGATGAGGTGCCACCGACACCACCGCCACTTACTGGTCAAATTGCAACAACGCCCAA
AAGTCAGTGCGACGGTGAACTACCGCGATCGAATACAGTGCCAACTCCGGCACGTTCCCTCGAGGGCTCCTGCCACTCATCTT
CGGCAACACCATGCTCCGCTTCTGGTGAACCCATCACCGTGCCATTAAGCCGTAAGCCTCCAGCAGTTAATCCAACCGTTAGA
AGCCCAGCAGCAAGTCAAAGCTCCGAGGTGTTCTTTGACAATTGTCAGAAGTTACTGGATAAATTCAATTACCCATGGGAGTT
GATGCCCCTAATGTATGTGATCTTAGAAAATGCCAACGTAGATATGCAGGAAGCAGCACGAAGGATTGAGGAAGGTCAATGTG
TTGTGAATGAATACACCCGAATGCACAATCTCAATATGTACGATGGAGCCGAACTGCGGGGTTCAACGAGACAATGTGGATGA
TAA
>PpadsxM_CDS [Phlebotomus papatasi] P. papatasi doublesexM CDS
ATGGTGTCGGAGCACAATACGTGGAGCGACGTGATGTCCAATTCGGACCTCACAGACTCGAAAAACGAGTTGTGCGGAGGAGC
CTCGAGCTCCAGCGGCAACTCATTGAGCCCGAGGACGCCACCAAATTGCGCCCGCTGCCGCAATCACGGCCTCAAGATAGCCC
TTAAGGGTCACAAACGCTACTGCAAGTACCGTTACTGCAACTGCGAAAAGTGTCGTCTAACGGCCGAACGTCAGCGGGTGATG
GCCTTGCAGACGGCCCTAAGACGTGCTCAGGCTCAGGACGAGGCACGTCAGATCTCCGCGGATGAGGTGCCACCGACGCCACC
GCCAGCCCCGTCCATTTGCACCCTTGCCACTCCTAAGAGTCAGTGCGATAGCGAACCACCGGCACCCATTACGGTGCCCACAC
CGGCACGTTCCCTAGAGGGTTCCTGCCACTCATCATCGGCCACACCCTGTTCTGCTTCCGGAGAACCCATCTCCGTGCCATCC
AGCCGCAAATTGGCCCCAGTCAATCCAACCATCAGAAATCCAACTGCAAGTCAAAGCTCCGAAGTCTTCTTTGACAATTGCCA
GAAATTGCTGGACAAATTCAATTATCCTTGGGAACTGATGCCCCTAATGTATGTGATCTTGGAAAATGCCCCGGACGTGAGGG
AAGCGGCACGGAGAATTGAAGAAGCTGCGAGTATAATAAAATGTCGATATGGACATGGACTCCCAATCCCAGCACACCAGTAC
TACACATACCTATCTACGGCGGCAGCGAGAGTGCAGACGTCGATATCCATATCCTTCGTACTACTACACATACTGGGCAGCCG
GTATACAAGTCCCTTCCTATATAACTTCTATAACGAGACAGTACTGTCGCCGCATTTGCACAGCAAGTACCTCAATTCGGAAA
GTACTCCTGCGGTACGGAATATATCGGAATCTCCGTCAGCCTATTCATCTCTGCCACTTCCTGCCGCTCTACAAATTACTCGT
CTGATACCAACGAATAGCTTATCTGGTGCATCGACGTAG
>LlodsxM_CDS [Lutzomyia longipalpis] L. longipalpis doublesexM CDS
ATGTCCAATTCGGACCTCACAGACTCGAAGAATGAGTTGTGCGGGGGAGCCTCGAGCTCCAGCGGCAACTCATTGAGCCCGAG
GACGCCGCCAAATTGCGCCCGCTGCCGCAATCACGGCCTCAAGATTGCCCTAAAGGGTCACAAGCGTTACTGCAAATTTCGCT
ACTGCAACTGCGAAAAGTGCCGCCTCACGGCCGAACGACAGCGGGTGATGGCCCTACAGACGGCCCTAAGACGTGCTCAGGCT

CAGGACGAGGCGCGTCAAATCTCCGCGGATGAGGTGCCACCGACACCACCGCCACTTACTGGTCAAATTGCAACAACGCCCAA
AAGTCAGTGCGACGGTGAACTACCGCGATCGAATACAGTGCCAACTCCGGCACGTTCCCTCGAGGGCTCCTGCCACTCATCTT
CGGCAACACCATGCTCCGCTTCTGGTGAACCCATCACCGTGCCATTAAGCCGTAAGCCTCCAGCAGTTAATCCAACCGTTAGA
AGCCCAGCAGCAAGTCAAAGCTCCGAGGTGTTCTTTGACAATTGTCAGAAGTTACTGGATAAATTCAATTACCCATGGGAGTT
GATGCCCCTAATGTATGTGATCTTAGAAAATGCCAACGTAGATATGCAGGAAGCAGCACGAAGGATTGAGGAAGCAATGGGAG
AGTGCAGACGTCGATATCCATATCCTTCGTACTACTACACATACTGGGCAGCCGGTAGTAGTCCCTACCTGTTCAATTACAAC
ATCCTCTCGTCGCAATTGCAAAATAAGAGCTTTAGCTGTGACAGTATTTCATCAACACGTGGCATCTCAGAGGCACCATCGTC
CTACCCCTCAATATCACTACCTGCCGCCGCCCTACAAATCAGACGTCTGATACCAACTGGTAACCTAGCCGGTGCATCGACGT
AG
>LlofruM_par_CDS [Lutzomyia longipalpis] L. longipalpis fruitlessM partial CDS
ATGATGTCACCACCGAATATGTACGGTGGTCCCTTTCAACCGTATCGGGGACAACCACACTCGTTGGTGTCCCCGCGCGAAGA
CAGCCCACCGACTTCGGTTTTAAACCTCAAGCGCTACACCACCAATGACCCTCCATCATCGCCGACCATACCTGTATCTGCGC
ATCATCACCTGCATCACACACGTGAGCAGGAGACATCGATGGCACATCAGCGACCGCTGTCAGCTCAGTCTCCACAGAGATAC
ACCACTGACCAAGGCAATATGGACCAGCAGTTCTGTCTTCGCTGGAATAATCATCCGACCAACTTGACTGGCGTGCTGACTTC
CTTGCTCCAGNGGGAGGCCCTCTGTGATGTCACACTGGCGTGC
>PdufruM_CDS [Phlebotomus duboscqi] P. duboscqi fruitlessM CDS
ATGATGACTACTCCGGACATCTTCGGCAGCCCGTTTCCGCCGTATAGGTGTCAGCCGTTGCCTGTACTTCCACCACGTGATGA
TAGTCCGCCAATGCCGGCGCTTGACCTTAAACGCTATCCCAGCACTGAGGTCCCACCGGCATCAACAACACCTGTCCGTTTAT
CTGAACGTGAAATGCCGGTGCCGCACCTGCGTCCATCTTCAGCACAAACACCACCTAGATACACCACTGACCAAGGCAATATG
GACCAGCAGTTCTGTCTTCGCTGGAATAATCATCCGACCAACTTGACTGGCGTGCTGACTTCTTTGCTCCAGAGGGAGGCCCT
CTGTGATGTTACATTGGCATGCGATGGTGGTGAAATTGTCAAGGCACATCAGACCATCCTGTCAGCATGCAGTCCATATTTTG
AAAGCATTTTCCTGCAGAACTCTCATCCGCATCCAATAATATATTTGAAAGATGTTAGATACTCAGAGATGCGCTCACTCCTT
GATTTCATGTACAAGGGTGAGGTGAACGTTGGCCAAAGATCGTTACCGACATTTCTAAAGACAGCCGAAAGTTTACAG
>PbefruM_CDS [Phlebotomus bergeroti] P. bergeroti fruitlessM CDS
ATGATGACTACTCCGGACATCTTCGGCAGCCCCTTTCCACCGTATAGGGGTCAGCCGTTGGCTGTACTTCCACCACGTGATGA
CAGTCCGCCAACGCCGGCACTTGACCTCAAACGCTATGCCAACACTGAGGTCCCTCCGGCATCAACAACACCTGTCGCACCAC
ACCTCCGTTTACCGCTGCATCATGCACATGAACGTGAAATGCCGATACCGCACCAGCGTCCATCTTCGGCACAAACACCACCT
AGATACACCACTGACCAAGGCAATATGGACCAGCAGTTCTGTCTTCGCTGGAATAATCATCCGACCAACTTGACTGGCGTGCT
GACTTCTTTGCTCCAGAGGGAGGCCCTCTGTGATGTTACATTGGCATGCGATGGTGGTGAAATTGTCAAGGCACATCAGACCA
TCCTGTCAGCATGCAGTCCATATTTTGAAAGCATTTTCCTGCAGAACTCTCATCCGCATCCAATAATATATTTGAAAGATGTT
AGATACTCAGAGATGCGCTCACTCCTTGATTTCATGTACAAGGGTGAGGTGAACGTTGGCCAAAGATCGTTACCGACATTTCT
AAAGACAGCCGAAAGTTTACAG
>PpafruM_CDS [Phlebotomus papatasi] P. papatasi fruitlessM CDS
ATGATGACTACTCCGGACATCTTCGGCAGCCCCTTTCCGCCGTATAGGTGTCAGCCGTTGGCTGTACTTCCACCACGTGATGA
TAGTCCGCCAATGCCGGCACTTGACCTCAAACGCTATCCCAGCACTGAGGTTCCTCCGGCATCAACAACACCTGTCCGTTTAC
CTGAACGTGAAATGCCGATGCCGCACCTGCGTCCATCTTCGGCACAAACACCACCTAGATACACCACTGACCAAGGCAATATG
GACCAGCAGTTCTGTCTTCGCTGGAATAATCATCCGACCAACTTGACTGGCGTGCTGACTTCTTTGCTCCAGAGGGAGGCCCT
CTGTGATGTTACATTGGCATGCGATGGTGGTGAAATTGTCAAGGCACATCAGACCATCCTGTCAGCATGCAGTCCATATTTTG
AAAGCATTTTCCTGCAGAACTCTCATCCGCATCCAATAATATATTTGAAAGATGTTAGATACTCAGAGATGCGCTCACTCCTT
GATTTCATGTACAAGGGTGAGGTGAACGTTGGCCAAAGATCGTTACCGACATTTCTAAAGACAGCCGAAAGTTTACAGGTACG
CGGTTTGACCGACAACAACAATATCAACTACCGACCAGAGAGCGACAGGGATCGCGATTCAGAAACGAATGCGAGTGGTGCTA
TGAAACATTATGATAAAACAGAACGGGACAGAGATCGCGACCGGGAGCGGCTGGACCGCGATCGTGAGGAGAATTCGGAGAGT
AAAGACCGCGATAGGGAGACACCTGTGGATCACCTGAGCAGCAGCAGCAGGAGCAGCAAGCGGAAACGTGAAAATTCATTAAA
CTGTGATAATTCAATGCGTGGCGCCAGTGTCCAAGAAAGGCATTATTCTCAGGATTCTCAGGCATCGTCGCATAGTAGTTATA
AATCCAGTCCATTGCCAAAATTAAATCCCCTGGAAGGAGAAGACACACGCCGAAATTCACCAGCGTTAAATGCCAGCGGCGCC
AATCAATCAGTTAGCATTAAACAAGAATTACCTGATATGGGCCATCATCCTGGTTTACCACCAGAATTACTTCCGCCCACTTC
AATGTCCCTGCATCCTGAGGATATGACAAGTCTACTTCCGGCTCATGGACTACAAGTGGAGACCTCGGAAAATGACTCGCAGC
ATCCTCAAATGGACCACAGTGATAATATCGATGGGCCGGGGGGGCATCGTCCACCTCCACCACTTCCACCGCCGCCACACCTC
CATCATCATGGACAGCACAGCAGTGGAGACGGAGAGTCAAAGCATCCACTGGTGCCAAGTCGATCGCGTTGTGGCGATGGCGG
CAGCAGTCGCGCCAGTCCCATCATGGGCGTCGTCGCCGCTGCCCTCCAGCACCATCAGCACCACCAGCATCAGATGTCCTACC
ACAATATGTTCTCGCCCACGAGAGAGCTGGCCGGCACCATGTGGCGATGTCGAACGTGCGGCAAGGAGGTCACCAACAGGTGG
CATCACTTCCATTCGCACACAGCCCAACGGAGCATGTGCCCCTACTGCCCGGCCACTTACAGTCGCATCGACACACTACGCTC
CCACCTCAGAGTAAAGCATCCCGATCGTCTGATCAAGAACTAG

7) Supplementary Methods References

Salvemini M, D'Amato R, Petrella V, Aceto S, Nimmo D, Neira M, et al. The orthologue of the fruitfly sex behaviour gene fruitless in the mosquito Aedes aegypti: evolution of genomic organisation and alternative splicing. PLoS One. 2013;8:e48554. doi:10.1371/journal.pone.0048554.

Salikhov K, Sacomoto G, Kucherov G. Using cascading bloom filters to improve the memory usage for de Brujin graphs. In: Lecture Notes in Computer Science (including subseries Lecture Notes in Artificial Intelligence and Lecture Notes in Bioinformatics). 2013. p. 364–76.

Grabherr MG, Haas BJ, Yassour M, Levin JZ, Thompson DA, Amit I, et al. Full-length transcriptome assembly from RNA-Seq data without a reference genome. Nat Biotechnol. 2011;29:644–52.

Haas BJ, Papanicolaou A, Yassour M, Grabherr M, Philip D, Bowden J, et al. reference generation and analysis with Trinity. Nat Protoc. 2014;8:1–43. doi:10.1038/nprot.2013.084.De.

Bolger AM, Lohse M, Usadel B. Trimmomatic: A flexible trimmer for Illumina sequence data. Bioinformatics. 2014;30:2114–20.
