# Supplementary material for: Identification of sex determination genes and their evolution in Phlebotominae sand flies (Diptera, Nematocera)
Source: BMC Genomics. 2019 Jun 25;20:522. doi: 10.1186/s12864-019-5898-4 (PMC6593557; doi:10.1186/s12864-019-5898-4)
Supplement: Supplementary file 8 — Figure S20. Multiple sequence alignment of sand fly TRA-2 proteins. (PDF 93 kb) [file 12864_2019_5898_MOESM8_ESM.pdf]

```
                           RS domain 1
LumTRA-2        ?--RNLFPQSSD-SSGYDR-RHSRTKKKRENGHHKKSSKS------RRRHRSRSHSK---  46
LneTRA-2        ?--RNLFPQSSD-SSGDDR-RYSRTKKKRENGHHKKSSKS------RRRHRSRSHSK---  46
PpeTRA-2A       --MTSDQKNSMSRSPSEGRSYILKGGSSRENNGYRRSDRSRSRR--RYRSRSRSHSQRRH  56
PbeTRA-2        MSTRTYYKNSMSRSPSEERHYISRAGSSRENGDYRKSRKSSSRSRGRYRSRSRSRS--RC  58
PpaTRA-2        MSTRTYYKNSMSRSPSEERHYISRAGSSRENGDYRKSRKSSSRSRARYRSRSRSRS--RS  58
PduTRA-2        MSTRTYYKNSMSRSPSEERHYISRAGSSRENGDYRKSRKSSSRSRGRYRSRSRSRS--RS  58
                    .   :* . * .  *    :  ..***. :::* :*      * * ****:*

                                                             RRM
LumTRA-2        -RRHYSRSPTRTTRKRHISRSPVPT--------YKKYEQPEEGRCLGVFGLSMHTTEKHV  97
LneTRA-2        -RRHYSRSPTRTIRKRHISRSPVPS--------YKKYEQPEEGRCLGVFGLSMHTTEKHV  97
PpeTRA-2A       RSRY-SGSPVRNDRKHSHSRSPHSSRRRHVG----SRDHPQQSRCLGVFGLSLITTKQQI  111
PbeTRA-2        EKRYRSKSPPRHDRKRSYSRSPNSSRRRHVASRVNSQDHPPKSRCLGVFGLSDATTEDQI  118
PpaTRA-2        EKRYRSRSPSRHDRKRSYSRSPNSSRRRHVASRVNSQDHPPKSRCLGVFGLSDATTEDQI  118
PduTRA-2        EKRHRSRSPSRHNRKRSYSRSPNSSRRRHVASRVNSQDHPPKSRCLGVFGLSDATTEDQI  118
                  *: * ** *  **:  ****  :          . ::* :.*********  **:.::
                                                              RNP-1

LumTRA-2        HEIFSKFGPIERTQIIVDSKTGRSRGYCFVYFENTEDAKVAKDQCTGIEIDDRRIRVDYS  157
LneTRA-2        HEIFAKFGPIERTQIIVDSKTGRSRGYCFVYFKNTEDAKVAKDQCTGIEIDDRRIRVDYS  157
PpeTRA-2A       NDIFSKFGRIKQIQIIMDAKTGRSRGFCFVYFADADDAKVARENCSGMEIDGRRIRVDYS  171
PbeTRA-2        YQIFSKFGTIHRTQIIMDAMTGCSRGFCFVYFANADDAKVAKDNCSGMELDGRRIRVDYS  178
PpaTRA-2        YQIFSKFGTIHRTQIIMDAMTGCSRGFCFVYFANADDAKVAKDNCSGMELDGRRIRVDYS  178
PduTRA-2        YQIFSKFGTIHRTQIIMDAMTGCSRGFCFVYFANADDAKVAKDNCSGMELDGRRIRVDYS  178
                 :**:*** *.: ***:*: ** ***:***** :::*****:::*:*:*:*.********
                                         RNP-2

                Linker Region      RS domain 2
LumTRA-2        LTARPHTPTPGIYMGKASQRCGD---SSTSRSNRYEDTS-YEKTRNRSPSPYKRR-RRSR  212
LneTRA-2        LTARPHTPTPGVYMGKASQRCGDRGDSSTSRSNRYEDSS-YEKTRNRSPSPYKRR-RRSR  215
PpeTRA-2A       ITERAHTPTPGKYMGVSTGRGREHNRSNRDDDYYYERESRSRYIERRSPSPYRRKRYRSR  231
PbeTRA-2        ITQRPHTPTPGVYMGQPTGRGRENRDRSRRN-----------------------------  209
PpaTRA-2        ITQRPHTPTPGVYMGQPTGRGRENRDRSRRN-----------------------------  209
PduTRA-2        ITQRPHTPTPGVYMGQPTGRGRENRDRSRRN-----------------------------  209
                :* * ****** ***  : *  :    .

LumTRA-2        SRSHSPRVRRYH       224
LneTRA-2        SRSYSPRVRRYH       227
PpeTRA-2A       SRSYSPRIRRYR       243
PbeTRA-2        -----------?       209
PpaTRA-2        -----------?       209
PduTRA-2        -----------?       209
```

Figure S20. Multiple sequence alignment of sand fly TRA-2 proteins. Sequence alignment of TRA-2 proteins of *Phlebotomus perniciosus* (PpeTRA2-A), *Phlebotomus papatasi* (PpaTRA2), *Phlebotomus bergeroti* (PbeTRA2), *Phlebotomus duboscqi* (PduTRA2), *Lutzomyia umbratilis* (LumTRA2-A) and *Lutzomyia neivai* (LneTRA2). The RNA recognition motif (RRM) is highlighted in black, RS domains are in light grey, and the linker region is in dark grey. Question marks indicate uncompleted sequences at N- or C- termini. Bold letters indicate amino acids identical between *P. perniciosus*, *L. umbratilis* and *L. neivai*. RNP-1 and RNP-2 indicate the positions of two ribonucleoprotein identifier sequences, which are highly conserved between RRM proteins. Note the high number of identical amino acids within the RRM domain, whereas both RS domains are less conserved but are abundant in arginine and serine. Gaps were introduced in the alignment to maximize similarity. The protein sequences alignment was performed using the Clustal-Omega software (1.2.4).
